# MiR-148a deletion protects from bone loss in physiological and estrogen-deficient mice by targeting NRP1

**DOI:** 10.1038/s41420-022-01261-5

**Published:** 2022-11-29

**Authors:** Bin Pan, Lin Zheng, Shijie Liu, Jiawei Fang, Chao Lou, Xingyu Hu, Lin Ye, Hehuan Lai, Jiawei Gao, Yejin Zhang, Kainan Ni, Dengwei He

**Affiliations:** 1 Department of Orthopedics, Lishui hospital affiliated to Zhejiang University School of Medicine, Zhejiang, China; 2 Key Laboratory of Imaging Diagnosis and Minimally Invasive Intervention Research of Zhejiang Province, Lishui hospital affiliated to Zhejiang University, Zhejiang, China

**Keywords:** Bone, Bone development

## Abstract

Bone metabolic homeostasis is largely dependent on the dynamic balance between osteoblasts and osteoclasts. MicroRNAs (miRNAs) play critical roles in regulating bone metabolism. In this study, we explored the role of a new miRNA (miR-148a) in osteoporosis. We compared the bone phenotype between miR-148a knockout (KO) mice and the wild-type (WT) littermates. We found miR-148a KO mice exhibited an increased bone mass phenotype and decreased osteoclastogenesis compared to the WT group. In vitro, miR-148a overexpression promoted osteoclastogenesis and bone resorption function. Mechanistically, NRP1 was identified as a novel direct target of miR-148a, and NRP1 silencing reversed the effect of miR-148a knockout. In OVX and calvarial osteolysis models, miR-148a KO protects mice against excessive bone resorption, while miR-148a agomiR/AAV-shNRP1 accelerates pathologic bone loss. Finally, the miR-148a level was found to be positively correlated with β-CTX in postmenopausal osteoporosis (PMOP) serum specimens. In summary, our findings revealed that miR-148a genetic deletion ameliorates bone loss under physiological and pathological conditions by targeting NRP1. In osteoclast-related bone metabolic diseases such as PMOP, miR-148a may be an attractive therapeutic target in the future.

## Introduction

Osteoporosis (OP) is defined as a severe metabolic disease characterized by systemic loss of bone mass and deterioration of strength and microarchitecture, which increase the propensity for fragility fractures [1]. Osteoclasts, the only specialized multinucleated cells that originate from the monocyte/macrophage lineage, are known to absorb the bone matrix by secreting acids and osteolytic enzymes [2]. Pathological stimulation, such as postmenopausal estrogen deficiency, bone tumor microenvironment, and aseptic inflammation, could facilitate the abnormal activation of osteoclasts [3]. Therefore, the exploration of the genes that target the biological functions of osteoclasts may be the key to preventing and treating bone metabolic diseases [4].

MicroRNAs (miRNAs), which are 22- to 24-nucleotide-long small noncoding RNAs, are involved in a wide range of biological processes [5, 6]. Insight into the roles of microRNAs in cancer, cardiovascular disease and infection is making microRNAs attractive tools and targets for therapeutic strategies. Several cancer therapeutics based on miRNA mimics or anti-miRNA have entered clinical development [7–9]. In maintaining bone homeostasis, miRNAs have been verified to regulate osteoblast or osteoclast differentiation and function, including miR-214 [10], miR-188 [11], miR-182 [12], miR-185 [13], miR-128 [14], and miR-29a [15]. These results suggest that studies on miRNAs targeting osteoblasts or osteoclasts can develop novel therapeutic strategies for osteoporosis. Although investigations of miRNAs in bone metabolism are expanding, the key miRNAs that regulate osteoclastogenesis and their functions in osteoporosis remain underexplored.

MiR-148a has been demonstrated to play roles in regulating both inflammasome signaling and immunological processes [16, 17]. Previous studies have demonstrated that miR-148a is involved in the inhibition of proliferation and apoptosis in osteosarcoma cells [18], as well as in the suppression of tumor invasion [19]. Recently, hsa-miR-148a was reported to have a key function in osteoarthritis and bone homeostasis [20]. Seeliger et al. identified five miRNAs that were highly expressed in the circulation and bone tissue of osteoporotic patients by miRNA PCR arrays, including miR-148a [21]. Cheng et al. reported that hsa-miR-148a mRNA was upregulated in the differentiation of RANKL-stimulated CD14^+^ (peripheral blood mononuclear cells) PBMCs toward osteoclast, and miR-148a directly targets V-maf musculoaponeurotic fibrosarcoma oncogene homolog B (MAFB) to promote osteoclastogenesis of PBMCs [22]. Despite these findings suggesting that miR-148a may be involved in the regulation of bone metabolic diseases, more advanced evidence is still required to further validate the function of miR-148a in physiological and pathological bone metabolism.

In this study, we generated miR-148a global knockout mice to investigate the effect of miR-148a on bone homeostasis. Our evidence showed that miR-148a is an important positive regulator of physiological and pathological bone loss by activating osteoclast differentiation. We demonstrated that NRP1 is a direct target of miR-148a and negatively regulates the NFATc1/c-Fos axis. Targeting miR-148a or its target may provide an attractive alternative treatment to prevent physiological and pathological bone loss.

## Results

### Global miR-148a deletion results in an increased bone mass phenotype

**Fig. 1:**
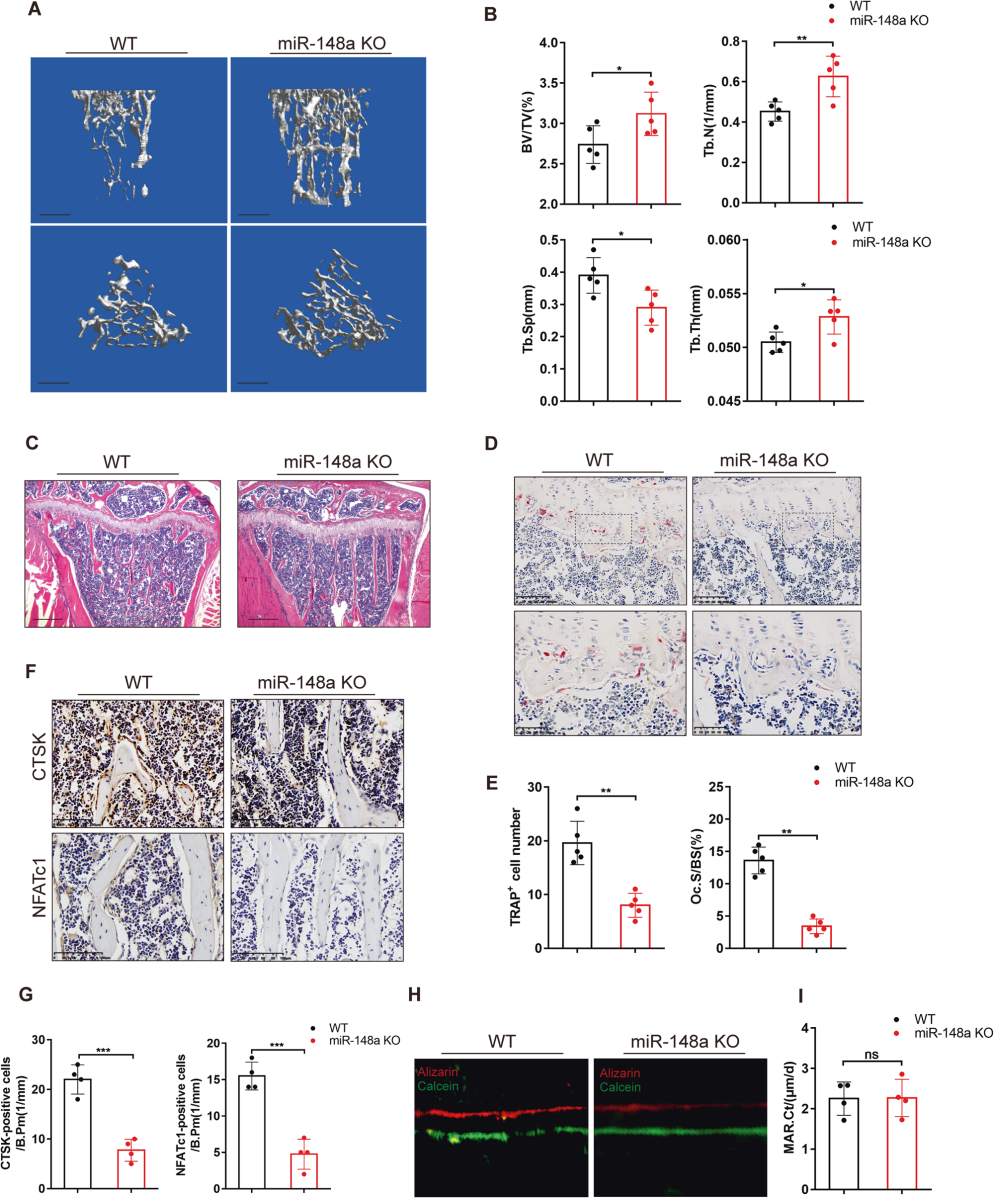
miR-148a knockout increased bone mass in mice under physiological conditions. **A** Representative 3D reconstructive microcomputed tomography (μCT) images of tibiae; Scale bar, 1 mm. **B** Quantitative analysis of the trabecular bone of 16-week-old female KO and WT mice; *n* = 5. **C** Representative H&E staining images of decalcified tibial bone sections from WT and KO mice; Scale bar, 500 μm. **D** Representative TRAP staining images of decalcified tibial bone sections from WT and KO mice; Scale bar, 100 μm. **E** Quantitative analysis of the number of TRAP-positive cells and the osteoclast surface/bone surface (Oc.S/BS); *n* = 5. **F** The expression of NFATc1 and CTSK in decalcified tibial bone sections from WT and KO mice, as detected by immunohistochemical staining; Scale bar, 100 μm. **G** Quantitative analysis of the number of positive cells in **F**; *n* = 4. **H** Representative images of calcein-alizarin red double fluorescent labeling demonstrated bone formation in mouse femur from 16-week-old female KO and WT mice; Scale bar, 5 μm. **I** Quantitative analysis of cortical bone mineral apposition rate (MAR) in **H**; *n* = 4. (All data are presented as mean ± SD; *p < 0.05; **p < 0.01; ***p < 0.001; ns no significance; compared to the littermate control group).

To investigate whether loss of miR-148a affected bone phenotype in vivo, we first constructed miR-148a global knockout mice (breeding information is presented in Fig. S1A. The littermates with a miR-148a KO genotype (hereafter referred to as wild type (WT)) were used as the controls. The KO mice were similar in appearance to WT mice at birth and after growth (Fig. S1C). The 3D reconstructive images based on high-resolution micro-CT demonstrated the increased bone mass phenotype in 16-week-old female KO mice when compared to WT controls (Fig. 1A). Quantitative bone parameter analysis revealed that miR-148a KO resulted in increases in Tb.N, Tb.Th and BV/TV and a decrease in Tb.Sp (Fig. 1B). H&E staining of proximal tibial bone sections showed that miR-148a KO mice had more trabecular bones (Fig. 1C).

Next, we assessed whether the high bone mass phenotype was caused by the activation of osteoblasts or the inhibition of osteoclasts. TRAP staining showed fewer TRAP-positive osteoclasts in the proximal tibial bone of 16-week-old miR-148a KO mice than in WT controls (Fig. 1D). Bone histomorphometric analysis revealed that miR-148a KO mice had fewer TRAP-positive cell numbers per bone surface and osteoclast-covered surface per bone surface (Oc.S/BS) than in the WT group (Fig. 1E). In addition, IHC staining confirmed that miR-148a deletion resulted in significant decreases in the expression levels of cathepsin K and NFATc1 (Fig. 1F, G), which were associated with osteoclast differentiation and bone resorption function. To analyze dynamic bone formation in mice, all mice were injected intraperitoneally with calcein green and Alizarin red at ten and three days prior to euthanasia. Interestingly, the mineral deposition rate (MAR) was not significantly influenced in miR-148a KO mice when compared to WT female mice (Fig. 1H, I).

Taken together, the above data suggested that global deletion of miR-148a leads to the increased bone mass phenotype in mice, possibly due to the inhibition of osteoclastogenesis, as osteoblast-mediated bone formation seems not to be altered in miR-148a KO mice.

### MiR-148a is a positive regulator of osteoclastogenesis in BMMs in vitro

**Fig. 2:**
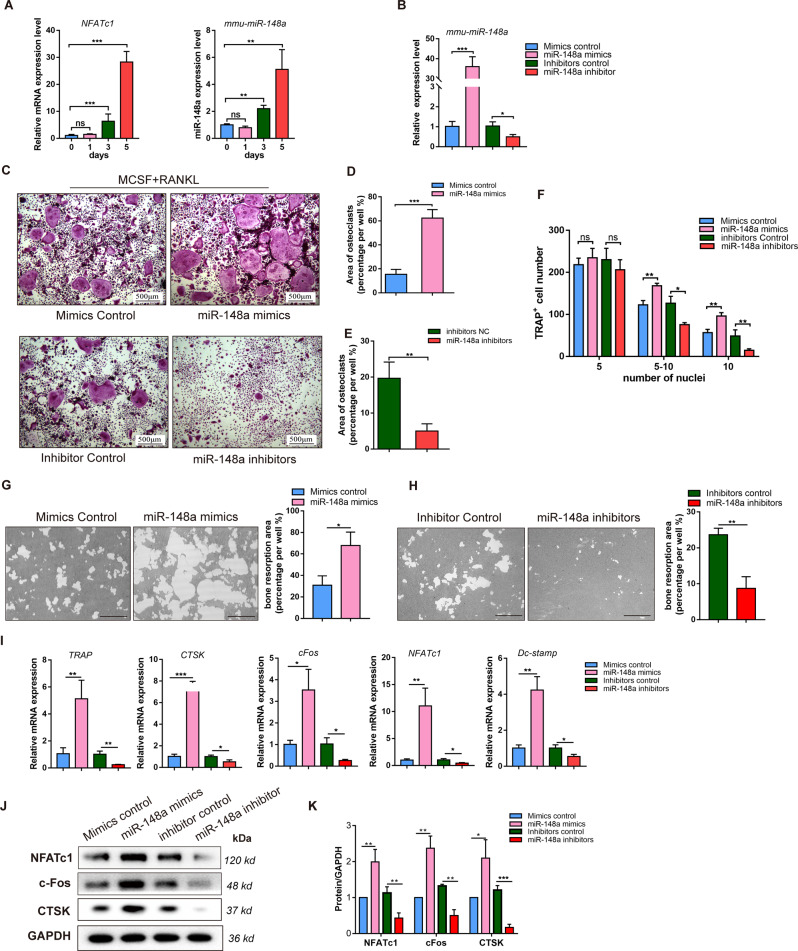
miR-148a is a positive regulator in osteoclastogenesis in vitro. **A** The mature mouse miR-148a and NFATc1 expression level were determined by RT-qPCR during RANKL-stimulated osteoclastogenesis. **B** The transfection efficiency of miR-148a mimics/inhibitors were evaluated by RT-qPCR. **C** The BMMs were transfected with miR-148a mimics/inhibitors and their corresponding control, and then cultured in osteoclast-inductive medium containing RANKL (50 ng/mL) for 4 days. TRAP staining was performed to observe the osteoclast formation; Scale bar, 500 μm. **D**–**F** Quantitative analysis of osteoclast area and TRAP-positive multinucleated cell numbers, *n* = 3. **G**, **H** The BMMs were seeded on hydroxyapatite-coated 96-well plate and transfected with miR-148a mimics/inhibitors and their corresponding control for 5 days. The transfection was re-performed again at the third day. Quantitative analysis of bone resorption pit area per view; Scale bar, 200 μm; *n* = 3. **I** The osteoclast-related gene expression of *Nfatc1*, *c-Fos*, *Ctsk*, *Trap*, and *Dc-stamp* were determined (day4) by RT-qPCR; *n* = 3. **J** The protein expression of NFATc1, c-Fos, CTSK were determined by western blot. **K** Quantitative analysis of protein expression in **J**; *n* = 3. (All data are presented as mean ± SD; *p < 0.05; **p < 0.01; ***p < 0.001; ns, no significance, compared to the corresponding control group).

We next examined whether miR-148a also regulated osteoclast differentiation and function in BMMs in vitro. Under RANKL stimulation, the expression levels of *miR-148a* and *Nfatc1* increased during the time course of osteoclast differentiation (0, 1, 3, and 5 days; Fig. 2A). Next, we constructed mimics/inhibitors to mimic miR-148a overexpression/silencing in vitro. Relative intracellular miR-148a levels in BMMs were determined by qRT-PCR after transfection with miR-148a mimics/inhibitors (Fig. 2B). More TRAP-positive multinucleated osteoclasts in the mimic-treatment group and decreased osteoclastogenesis in the inhibitor-treatment group than in each corresponding control group were observed (Fig. 2D–F), as evidenced by TRAP staining (Fig. 2C). Hydroxyapatite-coated plates were used to evaluate osteoclast bone resorption function. As expected, miR-148a mimic transfection significantly increased the osteoclast bone resorption area (Fig. 2G), and miR-148a inhibitor transfection impaired bone resorption function (Fig. 2H).

Meanwhile, genes or proteins associated with osteoclastogenesis, such as c-Fos and NFATc1 (initiating transcription of osteoclastogenesis genes); DC-STAMP (modulating cell–cell fusion); TRAP (involved in bone sialoprotein dephosphorylation); and CTSK (involved in osteoclastic bone resorption), were downregulated in the inhibitor-treated group, suggesting impaired osteoclastogenesis, but mimic treatment upregulated the expression levels of these genes (Fig. 2I–K). Taken together, these data suggest that miR-148a promotes osteoclastogenesis and bone resorption function in vitro in some way.

### MiR-148a deletion regulates bone homeostasis by inhibiting osteoclast activity

**Fig. 3:**
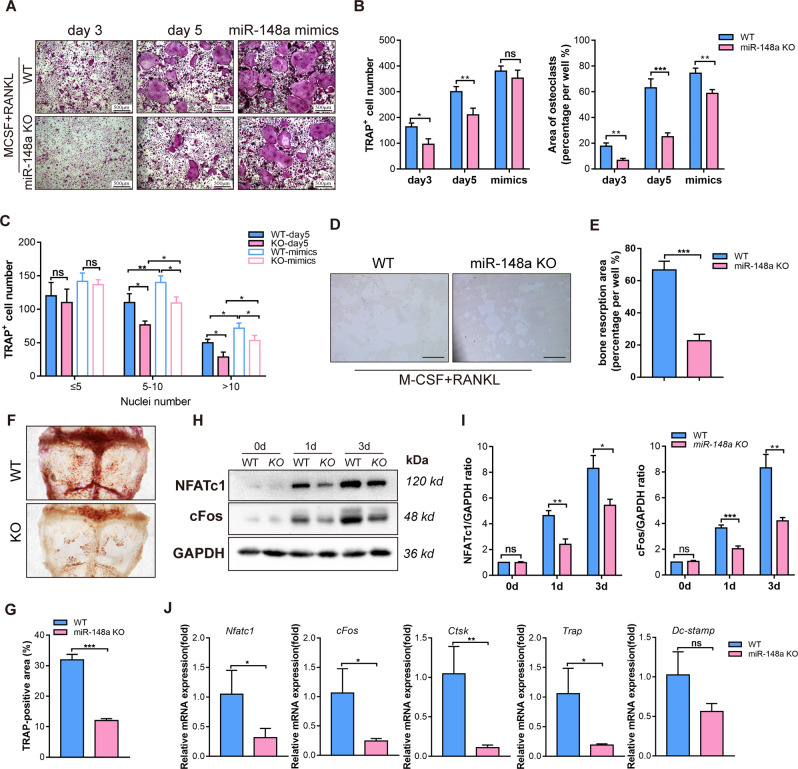
Deletion of miR-148a inhibits osteoclast formation and function in vitro. **A** TRAP staining to detect osteoclastogenesis of WT and miR-148a-deletion BMMs under RANKL stimulation; For mimics-supplementation group, the WT- and KO-derived BMMs were transfected with miR-148a mimics at day 1 and day 3, and harvested for TRAP staining at day 5; Scale bar, 500 μm. **B**, **C** Quantitative analysis of osteoclast area and TRAP-positive multinucleated cell numbers in **A**, *n* = 3. **D** WT-and KO-derived BMMs were seeded onto the hydroxyapatite-coated Osteo Assay plates and stimulated with RANKL (50 ng/mL) for 5 days; Scale bar, 200 μm. **E** Quantitative analysis of bone resorption area in **D**, *n* = 3. **F** Representative TRAP staining of the whole calvaria from WT and KO mice. **G** Quantitative analysis of TRAP-positive area in **F**; *n* = 3. **H** The protein expression of NFATc1 and c-Fos were determined by Western blot at 0, 1, and 3 days after RANKL stimulation. **I** Quantitative analysis of protein expression in **G**; *n* = 3. **J** Quantitative analysis of osteoclast-related gene expression (*Nfatc1*, *c-Fos*, *Ctsk*, *Trap*, and *Dc-stamp*) in WT and miR-148a KO osteoclasts by RT-qPCR; *n* = 3. (All data are presented as mean ± SD; *p < 0.05; **p < 0.01; ***p < 0.001; ns no significance, compared to the corresponding control group).

We isolated BMMs from WT and miR-148a KO mice to investigate the effect of miR-148a in osteoclast differentiation. The knockout efficiency is presented in Fig. S1B. In contrast to WT controls, miR-148a deletion in miR-148a KO mouse-derived BMMs dramatically decreased TRAP-positive multinucleated cell formation, as evidenced by TRAP staining (Fig. 3A), but this trend was diminished via supplementation with miR-148a mimics (Fig. 3A–C). Functionally, miR-148a deletion inhibited osteoclast resorption function in hydroxyapatite-coated plates, as shown in Fig. 3D, E. Compared to the calvaria tissue of WT mice, the whole calvaria tissue from KO mice showed lower TRAP activity (Fig. 3F, G). NF-κB and NFATc1/c-Fos signaling are critical regulators that initiates osteoclast differentiation signaling [23–25]. Above study revealed that miR-148a overexpression could promote NFATc1/c-Fos signaling activation in BMMs. To further confirm this result, we performed western blotting to analyze the effect of miR-148a KO on RANKL-induced NFATc1/cFos and NF-κB signaling. As shown in Fig. 3H, I, miR-148a KO significantly decreased the activation of RANKL-induced NFATc1/cFos signaling; Similarly, miR-148a KO significantly inhibited the activation of NF-κB signaling (Fig. S2A, B). RT–qPCR analysis further confirmed the above results, as shown in Fig. 3J.

In an in vitro osteoblast differentiation assay, we isolated BMSCs from the bone marrow of C57BL/6J mice. BMSCs were transfected with miR-148a agomir/antagomir, and the relative transfection efficiency was evaluated 7 days (Fig. S3A) and 21 days later (Fig. S3B). Our results revealed that miR-148a agomir or antagomir transfection had no obvious effect on alkaline phosphatase expression and osteoblast mineralization (Fig. S3C, D). Relevant mRNA expression levels of osteogenic specific genes (including *Runx2*, *Opn, Alp*, and *Ocn*) showed insignificant differences between agomir/antagomir treatment and their corresponding control group (Fig. S3E).

We hypothesized that miR-148a has a nonsignificant role in osteoblast differentiation. To confirm this hypothesis, we isolated miR-148 KO and corresponding WT mouse-derived BMSCs and cultured them in osteogenic medium for the indicated days. Our results revealed that miR-148a KO had no obvious effect on osteoblastogenesis (Fig. S4A, B) and osteogenic mineralization (Fig. S4C, D). Consistent with the above findings, analysis of the relevant osteogenic gene expression in Fig. S4E indicates that miR-148a plays a nonsignificant role in osteoblast differentiation. Collectively, our results suggest that miR-148a regulates bone homeostasis, mainly by affecting osteoclast activity.

### Identification of NRP1 as a direct target of miR-148a in BMMs

As mentioned previously, miRNAs mainly function at the posttranscriptional level through the RNA-induced silencing complex (RISC), which degrades target genes [26–28]. Based on this mechanism, we attempted to screen downregulated genes in osteoclastogenesis as the target of miR-148a, given that we found that miR-148a expression was upregulated during osteoclastogenesis.

**Fig. 4:**
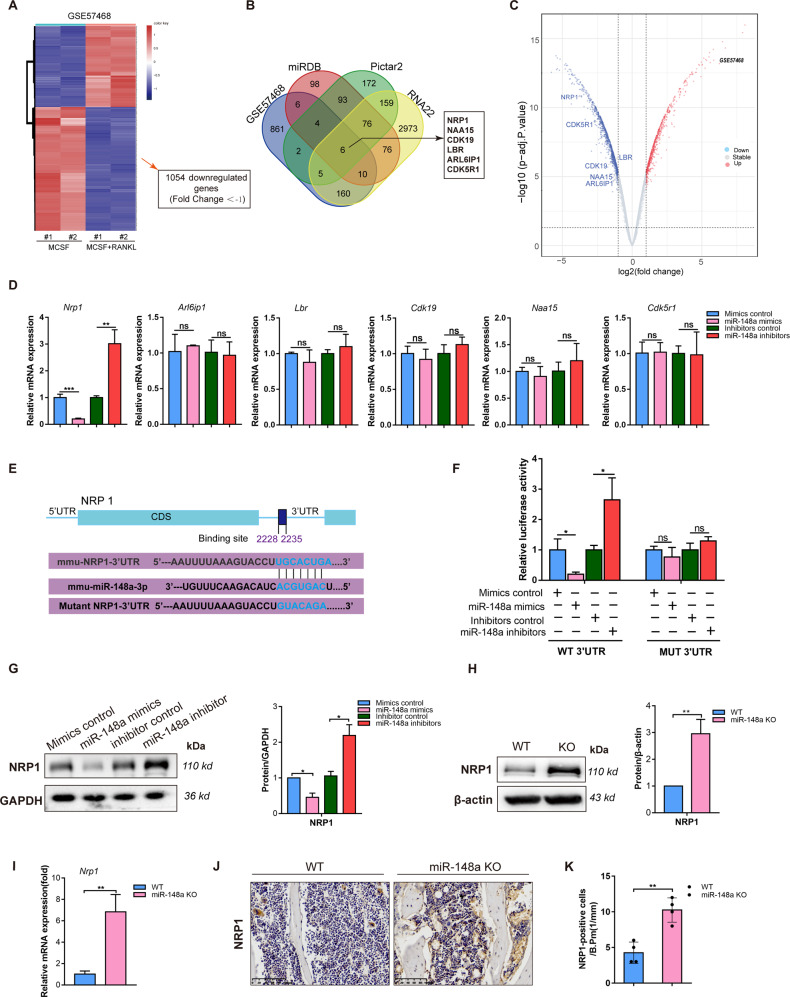
Identification of Nrp1 as a target of miR-148a. **A** Gene expression changes in mouse BMMs induced by RANKL for 3 days versus BMMs not induced by the RANKL from mRNA expression microarray GSE57468. **B** Venn diagram showing six candidate genes were computationally predicted to the target of miR-148a by different algorithms (miRDB、Pictar2、RNA22 and downregulated genes in GSE57468. **C** The heatmap displaying the candidate target genes expression in GEO: GSE57468. **D** The mRNA expression levels of 6 candidate genes in BMMs transfected with NC/ mimics or NC/inhibitors were evaluated by RT-qPCR, *n* = 3. **E** Schematic illustrations of the complementary sequence between miR-148a and 3′UTR (WT or Mutant) of Nrp1. **F** HEK-293 cells were co-transfected with miR-148a mimics (NC) or inhibitors (inhibitors NC) and luciferase reporter construct containing WT-3′ UTR or Mut-3′ UTR. The relative luciferase activities were detected, *n* = 3. **G** The protein expression of NRP1 in osteoclasts transfected with mimics/NC or inhibitors/NC were detected by Western blot, *n* = 3. **H** WT- and KO-derived BMMs were seeded on 6-well plates and cultured in the medium containing M-CSF and RANKL for 3 days. The protein expression of NRP1 were detected and quantified by Western blot, *n* = 3. **I** The mRNA expression levels of NRP1 in BMMs from WT- and KO-derived BMMs were evaluated by RT-qPCR, *n* = 3. **J** The expression of NRP1 in decalcified tibial bone sections from WT and KO mice, as detected by immunohistochemical staining, Scale bar, 100μm. **K** Quantitative analysis of the number of NRP1-positive cells in **J**, *n* = 4. (All data are presented as mean ± SD; *p < 0.05; **p < 0.01; ***p < 0.001; ns no significance, compared to the corresponding control group).

An analysis of the mRNA expression microarray from GSE57468 indicated that 1054 genes were downregulated during the differentiation from BMMs to osteoclasts upon M‐CSF and RANKL stimulation (|log2 (fold-change)| >1) (Fig. 4A). To further identify the target of miR148a, we searched the list of putative targets using miRWalk 2.0 [29], which includes prediction databases such as *Pictar2*, *miRDB* and *RNA22*. Then, we correlated the list of potential targets with the previously reported downregulated 1054 genes during osteoclastogenesis (assuming that the expression level of a given miRNA is inversely proportional to the expression level of its target). Finally, 6 candidate target genes were predicted to be potential targets of miR-148a in osteoclasts (Fig. 4B). The relevant downregulated genes were visualized in volcano maps (Fig. 4C). PCR analysis was performed to examine the expression of potential targets when transfected with miR-148a mimics/inhibitors. The results showed that *Nrp1* had the most significant correlation in BMMs when miR-148a mimics/inhibitors were transfected (Fig. 4D).

As predicted by the TargetScan database, miR-148 has a conserved binding site in the 3′-UTR of *Nrp1* (Fig. 4E). To determine whether miR-148a can directly interact with *Nrp1*, dual-luciferase assays were performed to predict the seed sequence binding sites responsible for the miRNA–mRNA interaction (Fig. 4F). 293T cells were then cotransfected with the miR-148a inhibitors/mimics and luciferase vectors containing the *Nrp1* WT 3′-UTR sequence or *Nrp1* mutant 3′-UTR sequences. The luciferase intensity was significantly downregulated by miR-148a mimics and upregulated by miR-148a inhibitors in the WT-3′UTR group. However, the results demonstrated that miR-148a mimic or inhibitor transfection had no effect on the mutant group. Besides, NRP1 protein levels were reduced after miR-148a overexpression in BMMs (Fig. 4G). As expected, the expression of NRP1 was markedly increased in miR-148a-deficient osteoclasts (Fig. 4H, I). Additionally, this effect was further supported by IHC staining, miR-148a deletion caused an increase in the expression of NRP1 (Fig. 4J, K). In conclusion, these results validate NRP1 as a direct target of miR-148a in BMMs.

### MiR-148a regulates osteoclastogenesis by modulating NRP1

**Fig. 5:**
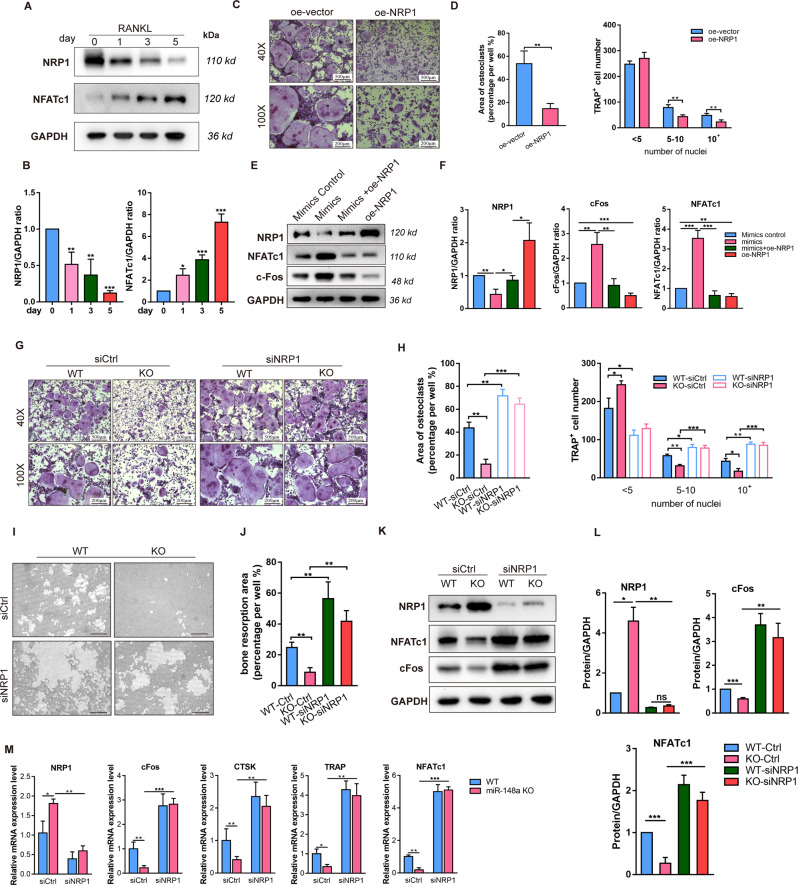
miR-148a inhibits osteoclast differentiation via targeting Nrp1. **A** The protein expression level of NRP1 and NFATc1 during the process of osteoclast differentiation (day 0, 1, 3, 5). **B** Quantitative analysis of the protein level of NFATc1 and NRP1 in **A**, compared with the BMMs without RANKL stimulation, *n* = 3. **C** BMMs were transfected with NRP1-overexpression plasmid and vector, and then cultured in osteoclast-inductive medium containing RANKL (50 ng/mL) for 4 days. TRAP staining was performed to observe the osteoclast formation; Scale bar, 500 μm. **D** Quantitative analysis of osteoclast area and TRAP-positive multinucleated cell numbers, *n* = 3. **E**, **F** The rescue experiments were performed in BMMs to identify the relationship between miR-148a and NRP1. Relative protein expression was quantified and presented in **F**, *n* = 3. **G** TRAP staining to detect osteoclastogenesis of WT and miR-148a KO cells transfected with siRNAs (siCtrl) or siRNA-targeted NRP1 (siNRP1) and cultured with RANKL for 5 days. The transfection mixture and osteoclast-inductive medium were replaced every 2 days until osteoclast could be observed; Scale bar, 500 μm. **H** Quantitative analysis of osteoclast area and TRAP-positive multinucleated cell numbers, *n* = 3. **I** Representative images for bone resorption by osteoclasts; Scale bar, 200 μm. **J** Quantitative analysis of bone resorption pit area per view; *n* = 3. **K** The protein expression of NRP1, NFATc1, and c-Fos in osteoclasts derived from WT and miR-148a KO mice that were transfected with the siCtrl or siNRP1. **L** Quantitative analysis of the protein level of NFATc1, c-Fos, and NRP1 in **K**; *n* = 3. **M** The osteoclast-related gene expression of *Nfatc1*, *c-Fos*, *Ctsk*, *Trap*, and *Dc-stamp* were determined by RT-qPCR; *n* = 3. (All data are presented as mean ± SD; *p < 0.05; **p < 0.01; ***p < 0.001; ns no significance, compared to the corresponding control group).

As previously reported, NRP1 has a critical role in osteoclast involving the formation of the Sema3A-NRP1-PlexinA1 complex. Consistent with previous studies, we further confirmed that NRP1 protein expression was downregulated during osteoclastogenesis (Fig. 5A, B). NRP1 was significantly overexpressed at mRNA and protein level by using lentivirus (Fig. S1D, E). TRAP-positive multinucleated cells were greatly reduced in osteoclasts that overexpressed NRP1 (Fig. 5C, D). The miR-148a mimics and oe-NRP1 lentivirus were then co-transfected into BMMs. Western blot results showed that oe-NRP1 partially reversed the miR-148a-induced upregulation of NFATc1/cFos (Fig. 5E, F).

Next, we performed rescue experiments with NRP1-targeted siRNA (siNRP1). The silencing efficiency of siNRP1 is shown in Fig. S1F, G. As shown in Fig. 5G, NRP1 silencing clearly reversed the inhibitory effect of miR-148a knockout in osteoclastogenesis when compared with the WT-derived BMMs, as evidenced by TRAP staining (Fig. 5H). In addition, NRP1 silencing rescued the impaired bone resorption function of mature osteoclasts in miR-148a-deletion osteoclasts, with a larger bone resorption area in hydroxyapatite-coated plates (Fig. 5I, J). After transfection with siNRP1, relevant osteoclastic markers, including NFATc1, c-Fos, CTSK, and TRAP, were upregulated at the mRNA and protein levels, which were inhibited by miR-148a deletion in osteoclasts before (Fig. 5K–M). Immunofluorescence (IF) revealed that suppression of NRP1 by RNAi significantly induced the nuclear transportation of NFATc1 (Fig. S2C). These results indicated that the miR-148a-NRP1 axis can regulate osteoclastogenesis by activating NFATc1/c-Fos signaling.

### Knockout or antagonism of miR-148a prevents Ti particle–induced osteolysis in vivo

**Fig. 6:**
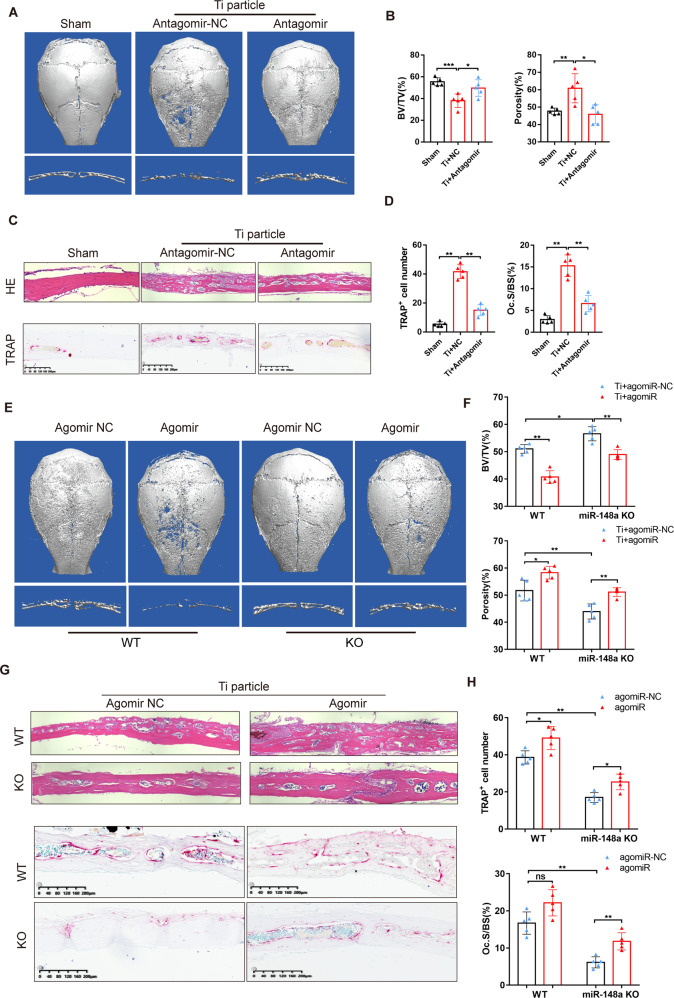
miR-148a antagomir or Knockout of miR-148a prevents Ti particle-induced osteolysis. **A** Representative μCT 3D reconstructed images of calvaria from sham-operated mice injected with PBS, and Ti-particle implantation mice injected with antagomir NC or antagomir-148a. **B** Quantitative analysis of bone volume/tissue volume (BV/TV) and percentage of porosity in **A**, *n* = 5. **C** H&E (upper panel) and TRAP staining (lower panel) of calvaria from the three groups mice; Scale bar, 200 μm. **D** Quantitative analysis of the number of TRAP-positive cells and the percentage of osteoclast surface per bone surface (Oc.S/BS), *n* = 5. **E** Representative μCT 3D reconstructed images of calvaria from four groups (WT and KO mice suffered from Ti-particle implantation were supplemented with agomiR-148a and agomiR-NC, respectively). **F** Quantitative analysis of bone volume/tissue volume (BV/TV) and percentage of porosity in **E**, *n* = 5. **G** H&E staining and TRAP staining of calvaria from the four groups mice; Scale bar, 200 μm. **H** Quantitative analysis of the number of TRAP-positive cells and the percentage of osteoclast surface per bone surface (Oc.S/BS), *n* = 5. (All data are presented as mean ± SD; *p < 0.05; **p < 0.01; ns, no significance, compared to the corresponding control group).

Our results obtained under physiological conditions showed that the miR-148a deletion has translational significance in maintaining bone homeostasis. Previous studies revealed that wear debris accumulated on the bone-graft surface and often triggered the aggregation of macrophages, eventually resulting in osteoclast activation and periprosthetic osteolysis (PPO) [30, 31]. We next explored whether antagonism of miR-148a could protect against Ti particle-induced osteolysis. AntagomiR-148a or NC transfection mixtures were injected into the subperiosteal region of calvaria as described previously. The expression of miR-148a in calvarial tissue was evaluated by RT–qPCR (Fig. S5A). μCT images demonstrated that mice subjected to Ti particle implantation and antagomir-NC treatment showed extensive calvarial destruction compared to the sham group (Fig. 6A). Quantitative analysis further revealed a significant increase in bone volume/total tissue volume (BV/TV) and a decrease in percentage porosity in the antagomir-148a group compared to the antagomir-NC group (Fig. 6B). H&E staining of calvarial sagittal sections showed that antagomir-148a treatment significantly reduced bone destruction caused by Ti particles (Fig. 6C). Meanwhile, antagomir-148a treatment significantly downregulated TRAP-positive cell number on the eroded bone surface compared with the control group, as evidenced by TRAP staining (Fig. 6C, D).

Given that miR-148a inhibition ameliorated Ti particle-induced bone destruction, we wondered whether restoring it in KO mice could reverse this phenotype in vivo (Fig. S5B). According to the reconstruction images of μCT, miR-148a KO (KO-NC group) ameliorated Ti particle-induced osteolysis compared to the WT-NC group. Meanwhile, supplementation with exogenous miR-148a (KO-agomir group) partially enhanced osteolysis compared with the KO-NC group (Fig. 6E). Quantitative analysis showed that the BV/TV was significantly decreased, while the porosities of the calvarial surface were increased compared to the KO-NC group (Fig. 6F).

The results of H&E staining demonstrated that miR-148a knockout significantly attenuated Ti particle-induced osteolysis, while supplementation with miR-148a agomir accelerated this process (Fig. 6G). TRAP staining revealed that genetic deletion of miR-148a markedly decreased the number of TRAP-positive cell; miR-148a supplementation increased the number of osteoclasts lining the eroded calvarial surface (Fig. 6G, H). These results further suggested that miR-148a knockout or antagonism could attenuate Ti particle-induced osteolysis by inhibiting osteoclastogenesis in vivo.

### Deletion of NRP1 reversed the bone-protective phenotype of miR-148a KO in estrogen-deficient mice

**Fig. 7:**
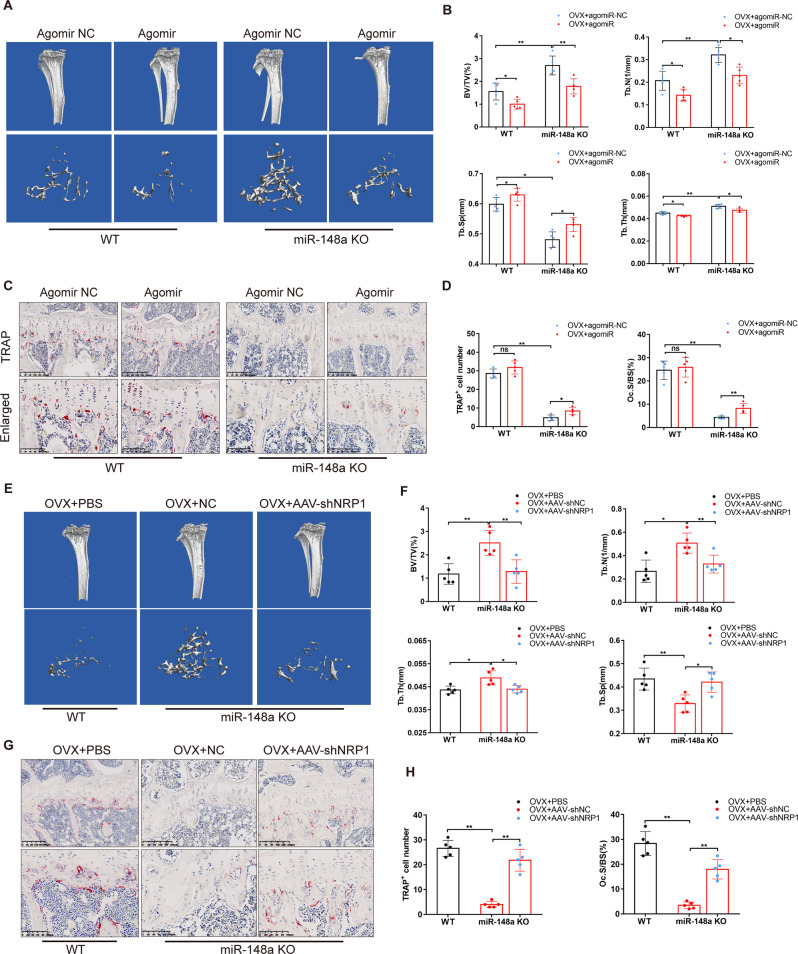
miR-148a supplementation or AAV-shNRP1 reverses the bone protection phenotype of KO mice. **A** Representative μCT 3D reconstructed images of tibiae from four groups (WT and KO mice suffered from OVX and supplemented with agomiR-148a and agomiR-NC, respectively). **B** Quantitative analysis of the trabecular bone parameters in **A**; *n* = 5. **C** Representative images of TRAP staining from four groups above, Scale bar, 200 μm. **D** Quantitative analysis of the number of TRAP-positive cells and the percentage of osteoclast surface per bone surface (Oc.S/BS); *n* = 5. **E** Representative μCT 3D reconstructed images of tibiae from three groups OVX mice (WT-PBS injection, KO-shNC injection, and KO-shNRP1 injection). **F** Quantitative analysis of trabecular bone parameters in **E**; *n* = 5. **G** TRAP staining of tibiae from the three groups above, Scale bar, 200 μm. **H** Quantitative analysis of the number of TRAP-positive cells and the percentage of osteoclast surface per bone surface (Oc.S/BS), *n* = 5. (All data are presented as mean ± SD; *p < 0.05; **p < 0.01; ns no significance, compared to the corresponding control group).

Next, we evaluated the effect of miR-148a deletion or supplementation with exogenous miR-148a agomir on ovariectomy (OVX) mice. The C57BL6/J mice were injected intravenously with miR-148a agomir or agomir-NC once a week for 5 weeks. The success of OVX surgery is marked by a significant loss of uterine weight (Fig. S5C). PCR results showed that miR-148a was upregulated in the bone tissues of agomir-injected mice compared to NC-injected mice (Fig. S5D). μCT images and quantitative analysis confirmed that the relevant parameters, including BV/TV, Tb.Th, and Tb.N, were significantly increased in NC-injected KO mice compared to NC-injected WT mice (Fig. 7A, B). However, supplementation with exogenous miR-148a decreased BV/TV, Tb.Th and Tb.N in the agomir-injected KO mice group compared to the NC-injected KO mice group (Fig. 7B). H&E staining further confirmed the above results (Fig. S5E), which could be attributed to the fact that miR-148a supplementation restored osteoclast numbers in KO mice (Fig. 7C, D).

Next, we validated the involvement of NRP1 as a direct target of miR-148a in the regulatory network of bone metabolism in vivo. All WT and KO mice subjected to OVX surgery (Fig. S6A) were divided into three groups, and the experimental protocol is described in detail in the Materials and Methods section. The AAV integration efficiency was confirmed by a green fluorescent protein (GFP) assay (Fig. S6B, C). μCT and H&E staining results showed that NRP1 knockdown attenuated the bone-protective effect of miR-148a KO in bone loss induced by estrogen deficiency (Figs. 7E, F and S6D). Similarly, AAV-shNRP1 injection increased the numbers of TRAP-positive cell, which were inhibited in miR-148a KO mice, as evidenced by TRAP staining (Fig. 7G, H). Collectively, we propose that miR-148a KO inhibits osteoclast formation and ameliorates bone loss by regulating the NRP1 signaling axis in an OVX-induced osteoporosis model.

### High miR-148a expression correlates with increased bone resorption in PMOP patients

**Fig. 8:**
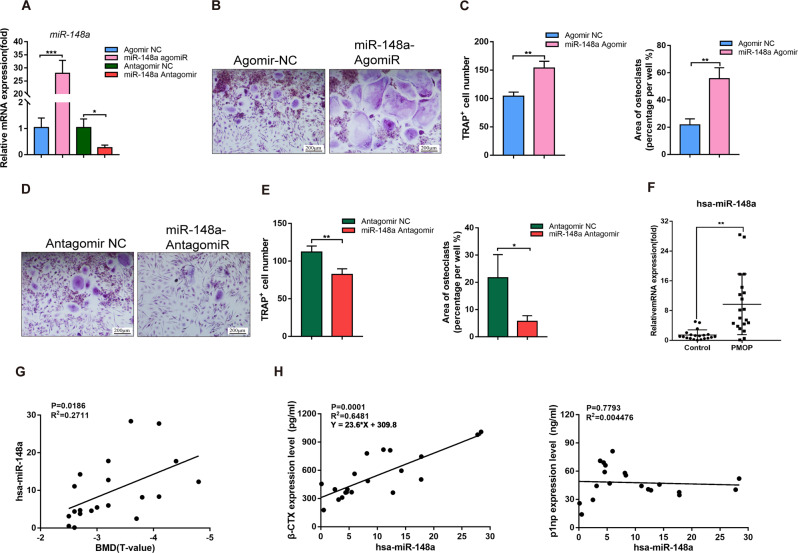
miR-148a levels positively correlate with increased bone resorption in PMOP patients. **A** PBMCs extracted from young volunteers were transfected with miR-148a agomiR/NC or antagomiR/NC. The expression of miR-148a levels were detected using RT-qPCR, *n* = 3. **B**, **D** PBMCs were transfected with agomiR/NC or antagomiR/NC, and then cultured in osteoclast-inductive medium containing recombinant human M-CSF (25 ng/mL) and recombinant human RANKL (50 ng/mL) for 10 days. TRAP staining was performed to observe the multinucleated osteoclasts formation; Scale bar, 200 μm. **C, E** Quantitative analysis of multinucleated cells area and TRAP-positive multinucleated cells numbers, *n* = 3. **F** Real-time qPCR analysis showed that miR-148a levels in whole serum from PMOP patients significantly increased. Human RUN6B was used as the internal control. *n* = 20 biologically independent samples. **G** Correlation analysis between the expression of miR-148a and BMD value in PMOP patients. *n* = 20 biologically independent samples. **H** Correlation analysis showed that the expression of miR-148a was positively associated with bone resorption makers β-CTX in PMOP patients. *n* = 20 biologically independent samples. (All data are presented as mean ± SD; *p < 0.05; **p < 0.01; ns no significance, compared to the corresponding control group).

We isolated hPBMCs from healthy population blood samples according to the manufacturer’s protocol. Next, we transfected miR-148a agomir/antagomir as well as the corresponding control into hPBMCs and observed osteoclast formation differences. The transfection efficiency of the miR-148a agomir/antagomir was evaluated by RT–qPCR, as shown in Fig. 8A. Our results show that miR-148a overexpression in PBMCs increased the number of TRAP-positive multinucleated cells and the average size of osteoclasts (Fig. 8B, C). While, the antagonism of miR-148a demonstrated a decreasing trend compared to the control group (Fig. 8D, E).

Next, we collected serum samples from patients clinically diagnosed with PMOP and the young population for PCR analysis. The remaining serum samples were performed with enzyme-linked immunosorbent assay (ELISA) assay to detect the expression of β-CTX and P1NP. The PMOP patients exhibited higher expression of hsa-miR-148a than the control population (Fig. 8F). In addition, we found that patients with lower BMD values tended to have higher miR-148a expression in the circulating blood (Fig. 8G); The expression of miR-148a were positively correlated with β-CTX expression levels in the serum of PMOP patients (Fig. 8H). Taken together, these results indicated that increased miR-148a expression in human serum was associated with increased bone resorption.

## Discussion

In this study, we provided substantial evidence revealed the role of miR-148a in osteoclast differentiation by utilizing miR-148a knockout mice. MiR-148a KO mice exhibited an increased bone mass phenotype under physiological and pathological conditions compared to the mice from WT littermates. MiR-148a overexpression accelerates osteoclast formation and resorption function in vitro. Meanwhile, NRP1 was verified as a novel target of miR-148a in osteoclastogenesis in response to RANKL stimulation. The AAV virus carrying a shRNA specific to NRP1 partially reversed the bone-protective phenotype in KO mice by promoting osteoclastogenesis when injected locally. The miR-148a-NRP1 axis acts as an important upstream signal to promote osteoclast differentiation by activating NFATc1/c-Fos signaling.

In recent years, the roles of miRNAs in the development and regulation of bone metabolic diseases have been widely noted [32]. Pathological conditions, such as PMOP and PPO, can lead to abnormal activation of osteoclast activity and induce bone loss [33, 34]. Several studies have proven that miRNAs targeting osteoclasts or osteoblasts show superior efficacy in the treatment of osteoporosis. For example, the miR-182-PKR-IFN-β axis was identified as a key positive signal for osteoclastogenesis, and miR-182 bone marrow-specific knockout protected mice from osteolysis induced by OVX or inflammatory arthritis [12]. In addition, miR-214 in circulation can target ATF4, an important osteogenic transcription factor, to inhibit bone formation [10, 35]. In addition, AAV-carried miR-214 weakened osteoclast activity in vivo and effectively prevented femoral head collapse in a rat model of osteonecrosis [36]. Based on previous research, increasing evidence imply that miR-148a may act as a critical regulator in the development of osteoporosis [21, 22, 37–39]. In this study, our data also showed that postmenopausal women had significantly higher miR-148a levels than premenopausal women (Fig. 8F). Furthermore, we found that miR-148a expression was positively correlated with a serum osteoporotic marker (β-CTX), emphasizing the potential of miR-148a to be a marker of bone resorption. However, these preliminary findings need to be validated using larger cohort studies.

MiR-148a, as an important epigenetic factor, is widely involved in many aspects of skeletal homeostasis, repair processes in vivo and macrophage differentiation in vitro [40–42]. Previously, miR-148a was reported to regulate inflammation-related diseases. Huang et al. reported that miR-148a acts as a downstream signal of Notch signaling, enhances M1 macrophage polarization and proinflammatory responses through PTEN/AKT-mediated upregulation of NF-κB signaling [16, 42]. Yao et al. found that PU.1 induced miR-148a transcription by binding to a conserved site in the miR-148a promoter; miR-148a deletion severely impaired monocyte differentiation through upregulation of target MAFB expression [43]. Osteoclasts are multinucleated giant cells that develop from monocyte/macrophage lineage precursors, which may indicate that miR-148a is a critical regulator in osteoclast differentiation. We observed a significant reduction of osteoclastogenesis in miR-148a-deficient BMMs, which may explain the phenotype of increased bone mass in KO mice. However, bone mass stabilization is established in a dynamic balance of bone formation and bone destruction. To date, the role of miR-148a in osteogenesis remains controversial. On one hand, Cheng et al. found that AntagomiR-148a administration decreased the mRNA expression of *Alp* in bone tissue and blood *Alp* expression in the circulation, but they failed to evaluate the role of miR-148a in osteoblast differentiation [22]. Huang et al. reported that miR-148a mimics promote osteoblast mineralization (ARS staining) by targeting the SMURF1/SMAD7/Bcl2 axis in BMSCs derived from rats with osteonecrosis of the femoral head (ONFH) [44]. On the other hand, Tian et al. reported that supplementation with miR-148a promoted adipogenic differentiation and blunted osteoblast differentiation by targeting Kdm6b in ST2 cell line [45]. Liu et al. showed that miR-148a may inhibit osteoblast differentiation by targeting P300 in the MC3T3-E1 cell line [46]. There seems to be controversy regarding the role of miR-148a in osteogenesis. This unstable experimental result that emerged within a well-established system suggests that the role of miR-148a on osteogenesis may not be significant. In our present study, we also noted that miR-148a genetic deficiency had insignificant trends in the differentiation and mineralization processes of osteoblasts. We inferred that miR-148a may be less expressed in osteoblasts, then the knockout of miR-148a does not have a significant effect on osteoblast differentiation. The bone-protective phenotype of miR-148a KO mice may be mainly caused by the inhibition of osteoclast activity. Although we used miR-148a KO mice, the effect of miR-148a on osteoblasts in different disease models needs to be further investigated. We assume that investigation of the role of miR-148a in osteoblast differentiation and bone loss by generating osteoblastic miR-148a knockout or transgenic mice (e.g. created by breeding miR-148a^flox/flox^ mice with a Prx1-Cre mice) will require further studies.

miRNAs function through their specific targets and target-mediated downstream pathways. Among the silencing complexes, Argonaute (AGO) protein, a crucial component of RISC, binds to miRNA and mediates mRNA degradation [26]. Target genes regulated by the same miRNA may also vary depending on cell and tissue type and various stress or disease environments, possibly due to different gene expression and regulatory profiles [47–49]. Neuropilin-1 (NRP1), a single-channel transmembrane protein, serves as a coreceptor for the class 3 semaphorin (Sema3) family, vascular endothelial growth factor (VEGF) and transforming growth factor–β (TGF-β). NRP1 plays multiple roles in axon guidance [50], angiogenesis [51], immunosuppression [52], cancer progression [53], and other physiological or pathological processes. Takegahara et al. reported that PlxnA1, a component of the NRP1 coreceptor complex, activates ITAM signaling by forming PlxnA1-TREM2-DAP12 complexes in response to ligands such as Sema6D, subsequently activating NFATc1/c-Fos signaling and promoting osteoclast differentiation [54]. Hiroshi et al. revealed that RANKL induced the formation of the PlxnA1-TREM2-DAP12 complex by downregulating the expression of NRP1. Briefly, the Sema3A-NRP1 axis competes with the TREM2-DAP12 complex for PlxnA1, thus inhibiting the costimulatory signaling induced by PlxnA1-TREM2-DAP12 in osteoclast [55]. Nrp1^Sema-^ mice have severe osteoporotic phenotypes, accompanied by an increase in osteoclast numbers [55]. In addition, Zhang et al. found that discoidin domain receptor 2 (DDR2), which belongs to the receptor tyrosine kinase (RTK) family, promotes NRP1 binding to PlxnA1, thereby isolating PlxnA1 from the PlxnA1-TREM2-DAP12 complex and leading to the inhibition of osteoclast formation [56]. In our present study, NRP1 overexpression suppressed the activation of NFATc1/c-Fos signaling in osteoclast. While, the inhibitory effect of miR-148a knockout on osteoclastogenesis and bone resorption function was reversed by using a specific small interfering RNA (siNRP1). Our experiments revealed NRP1could be a reliable target of miR-148a during osteoclast differentiation.

In postmenopausal patients with osteoporosis, osteoclasts are typically overactivated by estrogen deficiency, while osteoblasts are minimally affected [57]. Currently, widely used anti-osteoporosis drugs, including bisphosphonates, have the potential to increase the risk of fractures and osteonecrosis of the jaw with long-term use [58, 59]. Moreover, they can directly interfere with osteoblasts and affect the bone remodeling process [60]. MiR-148a appears to be a strong candidate for such drug development, as miR-148a reduces osteoclast-dominated bone resorption but does not affect osteoblast-mediated bone formation. We constructed two pathological models of bone loss triggered by osteoclast overactivation, including OVX and Ti particle-induced osteolysis. As expected, miR-148a KO significantly protected OVX- and Ti particle-induced bone loss, while treatment with chemically modified agomiR-148a led to the acceleration of bone loss.

In conclusion, we elucidated the important role of miR-148a in osteoclast differentiation and function by targeting NRP1 for the first time, which may provide a powerful and specific therapeutic target for the treatment of bone metabolic diseases caused by excessive bone resorption.

## Materials and methods

### Human serum specimen collection

This study was reviewed and approved by the medical ethics committee of the author’s hospital (Lishui Hospital affiliated of Zhejiang University). We collected serum specimens from 20 PMOP patients and 20 nonosteoporotic patients. Blood samples were taken from the patient’s intermedian cubital vein, and most samples were collected before 6:30 a.m. Then, the human samples were centrifuged to separate the serum and stored at -80 °C. All patients included in this study signed informed consent form prior to the beginning of the study. All patients met the inclusion criteria: including age >60 years; postmenopausal women with low-energy fractures; BMD less than −2.5 (T value) were defined as the PMOP group; or premenopausal women with acute fractures were defined as the control group. All subjects with rheumatoid arthritis (RA), diabetes, endocrine disease and malignancy were excluded from our study.

### Generation of miR-148a knockout mice

Mmu-miR-148a KO (C57BL/6 J genetic background) mice were constructed by the Model Animal Research Center (Nanjing University, Nanjing, China) using CRISPR/Cas9 technology. Genotype identification using DNA extracted from the tails and toes of mice showed a genomic loss in KO mice. The following primer sequences were used for genotyping: miR-148a-tF1: GCTTCTTGCCAAAGTTTAGTAACAG and miR-148a-tR1: TGCTCAATAAGTGAAGCCAGACTA. The C57BL/6J mice used in this study were supported by Silaike Experimental Animal Center, Shanghai. All mice were housed in a ventilated room with a natural light cycle and 23 °C environmental temperature and had free access to clean food and water. All animal experiments were approved by the Ethics Committee of Lishui Hospital affiliated with Zhejiang University) and were carried out according to the Guide for the Care and Use of Laboratory Animals from the National Institutes of Health.

### In vitro cell isolation and culture

For the osteoclastogenesis assays, we isolated mouse bone marrow macrophages (BMMs) from C57BL/6 J mouse femoral and tibial bone marrow flushes according to a previously published protocol [61]. Then, the isolated BMMs were cultured in warm complete α modification of Eagle’s medium (α-MEM) containing 10% fetal bovine serum (FBS) (Gibco, Gaithersburg, MD, United States), 25 ng/mL M-CSF (R&D system, Minneapolis, MN, USA) and 100 U/ml penicillin–streptomycin (Thermo Fisher, Waltham, Massachusetts, USA) for 4–5 days until they reached 90% confluence. Then, the BMMs were trypsinized and reseeded in 6-well culture plates at a density of 4×10^5^ cells/well (Western blot or qPCR) or 24-well plates at a density of 1×10^5^ cells/well (TRAP staining). BMMs were then induced to differentiate into osteoclasts in osteoclast-inductive medium containing M-CSF and 50 ng/mL RANKL (R&D Systems, Minneapolis, MN, USA) for 4–5 days until fused osteoclasts were observed.

Human peripheral blood mononuclear cells (hPBMCs) were isolated from human peripheral blood using Ficoll separation liquid (Solarbio Life Sciences, Beijing, China) and cultured in complete α-MEM containing recombinant human M-CSF (25 ng/mL) and recombinant human RANKL (50 ng/mL) for 10-12 days until fused osteoclasts formed. The complete medium was replaced every two days.

For the osteoblastogenesis assays, we isolated bone marrow mesenchymal stem cells (BMSCs) from the long bones (femoral and tibial marrow) of three- to four-week-old mice. Primary BMSCs were then cultured in α-MEM containing 10% FBS to remove redundant nonadherent and hematopoietic cells. Then, BMSCs were reseeded in the cell culture plates and cultured for 7 days or 21 days with osteogenic medium containing 50 μg/mL ascorbic acid (Sigma Aldrich, St Louis, USA), 5 mM β-glycerophosphate (Sigma Aldrich) and 100 nM dexamethasone. The osteogenic medium was replaced twice every three days.

### TRAP staining and bone resorption assay

Mature osteoclasts originated from BMMs (4–5 days) or PBMCs (10-12 days) were washed 3 times with phosphate-buffered saline (PBS) and immediately fixed in 4% paraformaldehyde (PFA) for 30 minutes at room temperature. Finally, these cells were stained with TRAP solution for one hour at 37 °C. Multinucleated cells with ≥3 nuclei were identified as TRAP-positive osteoclasts.

Osteoclastic bone resorption function was assayed by hydroxyapatite resorption assay. Briefly, we seeded BMMs (1×10^4^ cells/well) on hydroxyapatite-coated 96-well plates (Corning Inc., NY, USA) with complete α-MEM (α-MEM supplemented with 25 ng/mL M-CSF). After twenty-four hours, complete α-MEM was replaced with osteoclast-inductive medium (complete α-MEM supplemented with 50 ng/mL RANKL). The medium was replaced every two days until the osteoclasts were observed for 2 days. Finally, the wells were immersed in 5% sodium hypochlorite solution for 2 minutes and then rinsed with cold PBS until all cells were removed from the hydroxyapatite surface. The areas of bone resorption were photographed using a light microscope (Leica Microsystem, Germany) and were quantified using ImageJ software (NIH; Bethesda, MD, USA).

### Osteoblast differentiation and mineralization assays

For osteoblast alkaline phosphatase (ALP) staining, BMSCs were induced to differentiate into osteoblasts in osteogenic medium for 7 days. Osteogenic medium was changed twice every 3 days. Then, the plates were washed 3 times with clean PBS and fixed in 4% PFA for 30 minutes. Then, the cells were stained for 20-30 minutes using BCIP/NBT buffer (Beyotime, Shanghai, China). The percent of ALP-positive cells was observed by a Leica light microscope and quantified by ImageJ software.

To assess osteoblast mineralization function, BMSCs were induced to differentiate into osteoblasts in osteogenic medium for 21 days. Then, 1% Alizarin red S solution (Solarbio, Beijing, China) was added to each well for 30 minutes to visualize extracellular matrix calcium deposition. The representative mineralized nodules were photographed using a light microscope. Next, the degree of osteoblast mineralization was quantified by measuring the optical density (OD value) of the supernatants at 562 nm after distained with 10% acetylpyridinium chloride (Sigma-Aldrich, C9002-25G) for 1 h.

### RNA isolation and qRT–PCR

For quantification of relevant mRNA expression, total mRNA was extracted from osteoblasts and osteoclasts using the Ultrapure RNA Kit (CWBIO Inc., Beijing, China). Then, 1 μg of the total mRNA was reverse transcribed into cDNA using the HiFiScript cDNA synthesis kit (CWBIO Inc., Beijing, China) in a 20 μl reverse transcription system. Quantitative real-time PCR (qRT-PCR) analysis was performed on an ABI7500 Real-Time PCR system using the SYBR Green qPCR Master Mix kit (Yeasen, Shanghai, China). We normalized the relative gene expression to β-actin or GAPDH using the 2^-ΔΔCt^ method.

For quantification of relevant miRNA expression, total mRNA was isolated using the miRNA Purification Kit (CWBIO Inc., Beijing, China) and TRIzol reagent. Next, 1 µg of mRNA as template RNA was reverse transcribed into cDNA using the miRNA cDNA Synthesis Kit (Vazyme, Nanjing, China) in compliance with the manufacturer’s instructions. Quantitative RT-PCRs were performed using the miRNA Universal SYBR qPCR Master Mix (Vazyme, Nanjing, China) in a 20 μl reaction system. The conditions of the 40 cycles were 95 °C for 10 s and 60 °C for 30 s. Small nucleolar RNA (snRNA) U6 was used as an internal control. All primer sequences are listed in Supplementary Table S1.

### Western blotting

Total proteins were extracted according to a previously described protocol [62]. Equivalent amounts of protein were separated by 10% SDS–PAGE (20 μg per lane) and transferred to polyvinylidene difluoride (PVDF) membranes (Millipore, Bedford, MA, USA). After blocking with 5% skim milk (dissolved in TBST buffer), the membranes were incubated with primary antibody overnight at 4 °C, washed three times with TBST and incubated with horseradish peroxidase (HRP)-conjugated secondary antibody for one hour at room temperature. The primary antibodies were listed as follows: NFATc1 (1:1000; Santa Cruz, #sc-7294), CTSK (1:1000; Santa Cruz, #sc-48353), p-P65 (1:1000; CST, #3033), P65 (1:1000; CST, #8242), IkBα (1:1000; CST, #4814), c-Fos (1:1000; CST, #5348), GAPDH (1:3000; Abway, #AB0036), NRP1 (1:1000; Abcam, #81321), β-actin (1:3000; Abway, #AB0011). Finally, antibody activity was detected using ECL hypersensitive chemical luminescence reagents (Yeasen, Shanghai, China) and imaged by an Invitrogen iBright 1,500 system (Thermo Fisher Scientific Scientific). The band density was quantified using ImageJ software.

### Dual-luciferase assay

We constructed the pMIR-Report luciferase vector containing the binding site for miR-148a on NRP1’s 3′-UTR. To ensure the specificity of the binding target, the binding site for miR-148a was mutated from UGCACUGA to GUACAGA. We then constructed the NRP1-3′ UTR-WT-Luc plasmid and the NRP1-3′ UTR-Mut-Luc plasmid. Then, the WT-3′ UTR plasmid or Mut-3′ UTR plasmid was cotransfected with small RNA oligo into HEK-293T cells using Lipofectamine 2000 (Invitrogen). After 48 hours, the 293 T cells were collected and analyzed for luciferase activity using the dual-luciferase reporter assay system (Beyotime, Shanghai, China).

### Micro-computed tomography scanning

Mice were sacrificed via cervical dislocation, and the tibias of mice were fixed in 4% PFA for 48 h. The left tibia was scanned by a SkyScan 1275 micro-CT (Bruker, Billerica, MA, USA) with the following scanning conditions: 9 μm resolution, with an X-ray energy source set at 50 kV/75 μA. All raw data obtained from micro-CT scans were used for 3D reconstruction using SkyScan CTAn software. The counting started from 50 layers below the growth plate, and quantitative analysis of bone trabeculae was performed on 150 layers of scanned data. Parameters of interest, including trabecular thickness (Tb.Th), bone volume per tissue volume (BV/TV), numbers of trabeculae (Tb.N), and trabecular separation (Tb.Sp) were documented.

In the Ti-particle osteolysis model, we carefully removed the Ti-particle from the calvarial surface to avoid interference. For the quantitative analysis, we selected equal areas in the center of each calvaria as the regions of interest (ROI). Relevant parameters were analyzed using CTAn software, including bone volume/tissue volume (BV/TV) and the number of porosities in the ROI.

### Bone histomorphometric analysis and immunohistochemical staining

After the left tibias and calvarias were scanned by micro-CT, they were decalcified with 12.5% ethylenediaminetetraacetic acid (EDTA) for 2 weeks. The bone tissues were then dehydrated in different concentrations of ethanol and embedded in melted paraffin. All bone tissues were sectioned into 5-µm-thick sections by a microtome and then subjected to hematoxylin and eosin (H&E) and anti-tartrate acid phosphatase (TRAP) staining. Bone histomorphometry was evaluated in a blinded, nonbiased manner. The osteoclast numbers per bone surface and osteoclast-covered surface per bone surface (Oc.S/BS) in each sample were calculated.

For immunohistochemistry (IHC) analysis, sections were dewaxed, hydrated, subjected to antigen retrieval, permeabilized, blocked, incubated with primary antibody (NRP1, HUABIO, 1:100), (CTSK, Santa Cruz, 1:100), (NFATc1, Santa Cruz, 1:100), and then incubated with secondary antibody. Finally, color reactions were performed using diaminobenzidine (CWBIO, Shanghai, China) and hematoxylin counterstain.

### Cell transfection

miRNA mimics/inhibitors and agomiR/antagomiRs were supplied by *Gene Pharma* (Shanghai, China). For BMMs transfection, briefly, BMMs were seeded on 24-well plates at a density of 1 × 10^5^ cells/well and cultured in complete medium (containing 25 ng/mL M-CSF) until 80% confluence. Then, BMMs were transfected with either miR-148a mimics/control mimics or miR-148a inhibitors/inhibitors control using the Lipofectamine 3000 reagent (Invitrogen). Six hours later, the complete medium was replaced with osteoclast-inductive medium (containing 50 ng/mL RANKL). The transfection mixture and osteoclast-inductive medium were replaced every 2 days until osteoclasts could be observed. For mouse BMSCs and human-derived PBMCs, the cells were transfected with agomiR/antagomiR and their negative controls (NC). Then, the transfected cells were cultured in osteogenic medium or osteoclast differentiation medium for 21 or 10 days, respectively. The agomir/antagomir transfection were repeated every 5 days. The efficiency of transfection was evaluated by qRT–PCR.

### Plasmid construction, lentivirus preparation, and infection

The CDS sequence encoding NRP1 was synthesized and cloned into pCDH vector (CD510B, System Biosciences), and the new constructed plasmid was verified by sequencing and named as pCDH-NRP1 and pCDH empty vector. For lentivirus packing, the pCDH empty vector or pCDH-NRP1 and packaging plasmid mix (System Biosciences) were co-transfected into HEK293T cells with lipofectamine 2000. Supernatant was then harvested 48 hours after transfection and centrifuged to obtain lentiviral particles. For BMMs infection, the BMMs were seeded on six-well plates and cultured in complete medium (containing 25 ng/mL M-CSF) for 24 h. Then, the lentiviral solution was mixed with the medium and added to each well. The mRNA of NRP1 was extracted for qRT–PCR to evaluated the overexpression efficiency three days later.

For in vitro gene silencing experiments, mouse small interfering RNA (siRNA) was purchased from RiboBio (Guangzhou, China). We transfected siNRP1 and siCtrl into BMMs using Lipofectamine 2000 reagent. The detailed methods are described above. The knockdown efficiency of siNRP1 was evaluated by qRT–PCR and Western blot assays.

### Enzyme-linked immunosorbent assay

The bone turnover markers pro-collagen type I N-terminal peptide (P1NP) and β-C-terminal cross-linked telopeptides of type I collagen (β-CTX) were detected using ELISA kits (Roche Diagnostics GmbH, Penzberg, Germany).

### Ovariectomy-induced bone loss and supplementation with miR-148a in vivo

For in vivo miR-148a supplementation experiments, All WT and KO mice (10 weeks old) were subjected to OVX surgery and divided into 4 groups (WT-NC, WT-agomir, KO-NC, KO-agomir) (*n* = 5 per group). For the OVX procedure, all mice were subjected to general anesthesia, and bilateral ovariectomies were carried out through small dorsal incisions. The mice were injected intravenously with miR-148a agomir or NC as described previously [13, 63]. Briefly, miR-148a agomir (Gene Pharma, Shanghai, China) was dissolved in 50 μl of DEPC-treated water at a dose of 8ug/g and mixed with 25 μl of Entranster^TM^ In Vivo Transfection Reagent (Engreen, 18668-11-1, Beijing, China) and 100 μl of 10% glucose for 15 minutes at room temperature. These mice received miR-148a agomir mixture injection through the tail vein once a week for 5 weeks starting from the first week after OVX surgery. Six weeks after surgery, all mice were euthanized, and bilateral bone tissue was collected for the following experiments. The left bone tissue was collected for micro-CT scanning, bone histomorphometric analysis, and TRAP staining; The right bone tissue was collected for qRT-PCR analysis.

### Titanium-induced calvaria osteolysis model

Two independent sets of experiments were performed to verify the role of miR-148a in Ti particle-induced osteolysis. Titanium powder (purity 93%, <20 µm) was purchased from Alfa Aesar (#000681, Heysham, UK). First, the titanium powder was baked at 120 °C for 2 hours and then immersed in conFig.d 75% ethanol for 2 days to remove the endotoxin to the maximum extent possible [64]. The titanium powder was rewashed 3 times using sterile PBS, and the sterile PBS was readded to titanium powder to produce a 1 g/mL suspension. After the mice were subjected to general anesthesia, the skin covering the calvarium was shaved and disinfected, and then incised along the median line using a sharp surgical blade. Forty microliters (40 mg) of Ti particle suspension were spread around the sagittal line of the calvariae, and the incision was closed layer by layer. For the sham surgery group, the skin was incised, and 40 μl of sterile PBS was spread on the calvarial surface instead of Ti particles. In the first experiment, 24 C57BL/6 mice (12 weeks old, female) were divided into three groups (*n* = 8 per group): sham surgery + PBS treatment (sham group), Ti-particle implantation + NC injection, and Ti-particle implantation + antagomir injection. For the second experiment, all WT and KO mice were subjected to Ti particle implantation and divided into four groups (*n* = 8 per group): a WT-NC group, a WT-agomiR group, a KO-NC group and a KO-agomiR group. According to the manufacturer’s protocol, a mixture of miR-148a antagomir/agomir (5 μg/g) and Entranster^TM^ In Vivo Transfection Reagent was injected subcutaneously at a dose of 100 μl. Subcutaneous injection treatment was administered on Days 3, 7, and 11 after surgery. Calvarias were collected for RT–qPCR analysis (*n* = 3 per group), and micro-CT and histomorphometric analyses was performed on the remaining samples (*n* = 5 per group).

### AAV-shNRP1 treatment

The adeno-associated virus (AAV-9) vectors carrying shNRP1 or a negative control (shNC) were produced by Hanheng Biotechnology (Shanghai, China). All WT and KO mice (12 weeks old) were subjected to OVX surgery and divided into 3 groups (*n* = 8 per group): WT-PBS, KO-shNC, and KO-shNRP1. The KO mice received local injections of AAV-shNRP1 and AAV-shNC (20 μl) via syringe (Hamilton, Bonaduz, Switzerland) in the right tibial bone marrow cavity from the first week after surgery. WT mice received 20 μl PBS injection as a control group. GFP expression in vivo was detected after 4 weeks using an IVIS® Lumina Series III system (Perkin Elmer, USA). Six weeks after surgery, all mice were euthanized. The tibiae were subjected to micro-CT and histomorphometric analyses (*n* = 5 per group).

### Statistical analysis

Statistical analysis was performed by using Prism 7.0 (GraphPad Software, San Diego, CA, USA). Data are expressed as mean ± standard deviation (SD). The comparisons between experimental and control groups were made by unpaired Student’s *t* test or one-way ANOVA with Tukey’s test. *P* < 0.05 was considered statistically significant.

## Supplementary information

Supplementary Figures

Supplementary Tables

Original data files

## Data Availability

The date used to support the findings of this study are available from the corresponding author or the Supplementary Materials section upon request.

## References

[1] Compston JE, McClung MR, Leslie WD (2019). Osteoporosis. Lancet.

[2] Søe K, Delaisse JM, Borggaard XG (2021). Osteoclast formation at the bone marrow/bone surface interface: Importance of structural elements, matrix, and intercellular communication. Semin Cell Dev Biol.

[3] Novack DV, Teitelbaum SL (2008). The osteoclast: friend or foe?. Annu Rev Pathol.

[4] Gennari L, Merlotti D, Falchetti A, Eller Vainicher C, Cosso R, Chiodini I (2020). Emerging therapeutic targets for osteoporosis. Expert Opin Ther Targets.

[5] Rigoutsos I, Furnari F (2010). Gene-expression forum: decoy for microRNAs. Nature.

[6] Kloosterman WP, Plasterk RH (2006). The diverse functions of microRNAs in animal development and disease. Dev Cell.

[7] Rokavec M, Öner MG, Li H, Jackstadt R, Jiang L, Lodygin D (2014). IL-6R/STAT3/miR-34a feedback loop promotes EMT-mediated colorectal cancer invasion and metastasis. J Clin Invest.

[8] Misso G, Di Martino MT, De Rosa G, Farooqi AA, Lombardi A, Campani V (2014). Mir-34: a new weapon against cancer?. Mol Ther Nucleic Acids.

[9] van Zandwijk N, Pavlakis N, Kao S, Clarke S, Lee A, Brahmbhatt H, et al. MesomiR 1: a phase I study of TargomiRs in patients with refractory malignant pleural mesothelioma (MPM) and lung cancer (NSCLC). Ann. Oncol. 2015;26:ii16.

[10] Wang X, Guo B, Li Q, Peng J, Yang Z, Wang A (2013). miR-214 targets ATF4 to inhibit bone formation. Nat Med.

[11] Li CJ, Cheng P, Liang MK, Chen YS, Lu Q, Wang JY (2015). MicroRNA-188 regulates age-related switch between osteoblast and adipocyte differentiation. J Clin Invest.

[12] Inoue K, Deng Z, Chen Y, Giannopoulou E, Xu R, Gong S (2018). Bone protection by inhibition of microRNA-182. Nat Commun.

[13] Cui Q, Xing J, Yu M, Wang Y, Xu J, Gu Y (2019). Mmu-miR-185 depletion promotes osteogenic differentiation and suppresses bone loss in osteoporosis through the Bgn-mediated BMP/Smad pathway. Cell Death Dis.

[14] Shen G, Ren H, Shang Q, Zhang Z, Zhao W, Yu X (2020). miR-128 plays a critical role in murine osteoclastogenesis and estrogen deficiency-induced bone loss. Theranostics.

[15] Lian WS, Ko JY, Chen YS, Ke HJ, Hsieh CK, Kuo CW (2019). MicroRNA-29a represses osteoclast formation and protects against osteoporosis by regulating PCAF-mediated RANKL and CXCL12. Cell Death Dis.

[16] Gonzalez-Martin A, Adams BD, Lai M, Shepherd J, Salvador-Bernaldez M, Salvador JM (2016). The microRNA miR-148a functions as a critical regulator of B cell tolerance and autoimmunity. Nat Immunol.

[17] Dai Y, Wang S, Chang S, Ren D, Shali S, Li C (2020). M2 macrophage-derived exosomes carry microRNA-148a to alleviate myocardial ischemia/reperfusion injury via inhibiting TXNIP and the TLR4/NF-κB/NLRP3 inflammasome signaling pathway. J Mol Cell Cardiol.

[18] Bhattacharya S, Chalk AM, Ng AJ, Martin TJ, Zannettino AC, Purton LE (2016). Increased miR-155-5p and reduced miR-148a-3p contribute to the suppression of osteosarcoma cell death. Oncogene.

[19] Li J, Song Y, Wang Y, Luo J, Yu W (2013). MicroRNA-148a suppresses epithelial-to-mesenchymal transition by targeting ROCK1 in non-small cell lung cancer cells. Mol Cell Biochem.

[20] Vonk LA, Kragten AH, Dhert WJ, Saris DB, Creemers LB (2014). Overexpression of hsa-miR-148a promotes cartilage production and inhibits cartilage degradation by osteoarthritic chondrocytes. Osteoarthr Cartil.

[21] Seeliger C, Karpinski K, Haug AT, Vester H, Schmitt A, Bauer JS (2014). Five freely circulating miRNAs and bone tissue miRNAs are associated with osteoporotic fractures. J Bone Min Res.

[22] Cheng P, Chen C, He HB, Hu R, Zhou HD, Xie H (2013). miR-148a regulates osteoclastogenesis by targeting V-maf musculoaponeurotic fibrosarcoma oncogene homolog B. J Bone Min Res.

[23] Novack DV (2011). Role of NF-κB in the skeleton. Cell Res.

[24] Aliprantis AO, Ueki Y, Sulyanto R, Park A, Sigrist KS, Sharma SM (2008). NFATc1 in mice represses osteoprotegerin during osteoclastogenesis and dissociates systemic osteopenia from inflammation in cherubism. J Clin Invest.

[25] Lorenzo J (2017). The many ways of osteoclast activation. J Clin Invest.

[26] Fabian MR, Sonenberg N (2012). The mechanics of miRNA-mediated gene silencing: a look under the hood of miRISC. Nat Struct Mol Biol.

[27] Eulalio A, Huntzinger E, Izaurralde E (2008). Getting to the root of miRNA-mediated gene silencing. Cell.

[28] Mathonnet G, Fabian MR, Svitkin YV, Parsyan A, Huck L, Murata T (2007). MicroRNA inhibition of translation initiation in vitro by targeting the cap-binding complex eIF4F. Science.

[29] Sticht C, De La Torre C, Parveen A, Gretz N (2018). miRWalk: An online resource for prediction of microRNA binding sites. PLoS ONE.

[30] Gallo J, Vaculova J, Goodman SB, Konttinen YT, Thyssen JP (2014). Contributions of human tissue analysis to understanding the mechanisms of loosening and osteolysis in total hip replacement. Acta Biomater.

[31] Bi Y, Van De Motter RR, Ragab AA, Goldberg VM, Anderson JM, Greenfield EM (2001). Titanium particles stimulate bone resorption by inducing differentiation of murine osteoclasts. J Bone Joint Surg Am.

[32] Inoue K, Ng C, Xia Y, Zhao B (2021). Regulation of osteoclastogenesis and bone resorption by miRNAs. Front Cell Dev Biol.

[33] Purdue PE, Koulouvaris P, Potter HG, Nestor BJ, Sculco TP (2007). The cellular and molecular biology of periprosthetic osteolysis. Clin Orthop Relat Res.

[34] Pharmacotherapy for Postmenopausal Osteoporosis. JAMA. 2021;325:1888-9. 10.1001/jama.2020.13841 33974021

[35] Li D, Liu J, Guo B, Liang C, Dang L, Lu C (2016). Osteoclast-derived exosomal miR-214-3p inhibits osteoblastic bone formation. Nat Commun.

[36] Wang C, Sun W, Ling S, Wang Y, Wang X, Meng H (2019). AAV-Anti-miR-214 prevents collapse of the femoral head in osteonecrosis by regulating osteoblast and osteoclast activities. Mol Ther Nucleic Acids.

[37] Yuan H, Xu X, Feng X, Zhu E, Zhou J, Wang G (2019). A novel long noncoding RNA PGC1β-OT1 regulates adipocyte and osteoblast differentiation through antagonizing miR-148a-3p. Cell Death Differ.

[38] Jones TL, Esa MS, Li KHC, Krishnan SRG, Elgallab GM, Pearce MS (2021). Osteoporosis, fracture, osteoarthritis & sarcopenia: A systematic review of circulating microRNA association. Bone.

[39] Gu H, Wu L, Chen H, Huang Z, Xu J, Zhou K (2019). Identification of differentially expressed microRNAs in the bone marrow of osteoporosis patients. Am J Transl Res.

[40] Hensley AP, McAlinden A (2021). The role of microRNAs in bone development. Bone.

[41] Bai X, Li J, Li L, Liu M, Liu Y, Cao M (2020). Extracellular vesicles from adipose tissue-derived stem cells affect notch-miR148a-3p axis to regulate polarization of macrophages and alleviate sepsis in mice. Front Immunol.

[42] Huang F, Zhao JL, Wang L, Gao CC, Liang SQ, An DJ (2017). miR-148a-3p mediates notch signaling to promote the differentiation and M1 activation of macrophages. Front Immunol.

[43] Meng Y, Li J, Ye Z, Yin Z, Sun Q, Liao Z, et al. MicroRNA-148a facilitates inflammatory dendritic cell differentiation and autoimmunity by targeting MAFB. JCI Insight. 2020;5:e133721. 10.1172/jci.insight.133721 PMC7205423 32213710

[44] Huang S, Li Y, Wu P, Xiao Y, Duan N, Quan J (2020). microRNA-148a-3p in extracellular vesicles derived from bone marrow mesenchymal stem cells suppresses SMURF1 to prevent osteonecrosis of femoral head. J Cell Mol Med.

[45] Tian L, Zheng F, Li Z, Wang H, Yuan H, Zhang X (2017). miR-148a-3p regulates adipocyte and osteoblast differentiation by targeting lysine-specific demethylase 6b. Gene.

[46] Liu N, Sun Y (2021). microRNA-148a-3p-targeting p300 protects against osteoblast differentiation and osteoporotic bone reconstruction. Regen Med.

[47] Bartel DP (2004). MicroRNAs: genomics, biogenesis, mechanism, and function. Cell.

[48] Singh RP, Massachi I, Manickavel S, Singh S, Rao NP, Hasan S (2013). The role of miRNA in inflammation and autoimmunity. Autoimmun Rev.

[49] Jonas S, Izaurralde E (2015). Towards a molecular understanding of microRNA-mediated gene silencing. Nat Rev Genet.

[50] Chen C, Li M, Chai H, Yang H, Fisher WE, Yao Q (2005). Roles of neuropilins in neuronal development, angiogenesis, and cancers. World J Surg.

[51] Koch S (2012). Neuropilin signalling in angiogenesis. Biochem Soc Trans.

[52] Sarris M, Andersen KG, Randow F, Mayr L, Betz AG (2008). Neuropilin-1 expression on regulatory T cells enhances their interactions with dendritic cells during antigen recognition. Immunity.

[53] Chuckran CA, Liu C, Bruno TC, Workman CJ, Vignali DA. Neuropilin-1: a checkpoint target with unique implications for cancer immunology and immunotherapy. J Immunother Cancer. 2020;8:e000967. 10.1136/jitc-2020-000967 PMC7368550 32675311

[54] Takegahara N, Takamatsu H, Toyofuku T, Tsujimura T, Okuno T, Yukawa K (2006). Plexin-A1 and its interaction with DAP12 in immune responses and bone homeostasis. Nat Cell Biol.

[55] Hayashi M, Nakashima T, Taniguchi M, Kodama T, Kumanogoh A, Takayanagi H (2012). Osteoprotection by semaphorin 3A. Nature.

[56] Zhang Y, Su J, Wu S, Teng Y, Yin Z, Guo Y (2015). DDR2 (discoidin domain receptor 2) suppresses osteoclastogenesis and is a potential therapeutic target in osteoporosis. Sci Signal.

[57] Gossiel F, Altaher H, Reid DM, Roux C, Felsenberg D, Glüer CC (2018). Bone turnover markers after the menopause: T-score approach. Bone.

[58] Russell RG, Watts NB, Ebetino FH, Rogers MJ (2008). Mechanisms of action of bisphosphonates: similarities and differences and their potential influence on clinical efficacy. Osteoporos Int.

[59] Lambrinoudaki I, Christodoulakos G, Botsis D (2006). Bisphosphonates. Ann N Y Acad Sci.

[60] Im GI, Qureshi SA, Kenney J, Rubash HE, Shanbhag AS (2004). Osteoblast proliferation and maturation by bisphosphonates. Biomaterials.

[61] Cao X, He W, Rong K, Xu S, Chen Z, Liang Y (2021). DZNep promotes mouse bone defect healing via enhancing both osteogenesis and osteoclastogenesis. Stem Cell Res Ther.

[62] Pan B, Zheng L, Fang J, Lin Y, Lai H, Gao J (2021). Azilsartan suppresses osteoclastogenesis and ameliorates ovariectomy-induced osteoporosis by inhibiting reactive oxygen species production and activating Nrf2 signaling. Front Pharmacol.

[63] Li H, Xie H, Liu W, Hu R, Huang B, Tan YF (2009). A novel microRNA targeting HDAC5 regulates osteoblast differentiation in mice and contributes to primary osteoporosis in humans. J Clin Invest.

[64] Zhang L, Yang Y, Liao Z, Liu Q, Lei X, Li M (2020). Genetic and pharmacological activation of Hedgehog signaling inhibits osteoclastogenesis and attenuates titanium particle-induced osteolysis partly through suppressing the JNK/c-Fos-NFATc1 cascade. Theranostics.

