# Supplementary material for: MiR-148a deletion protects from bone loss in physiological and estrogen-deficient mice by targeting NRP1
Source: Cell Death Discov. 2022 Nov 29;8:470. doi: 10.1038/s41420-022-01261-5 (PMC9708754; doi:10.1038/s41420-022-01261-5)

**Supplementary Materials**

**Supplementary Fig S1: Gene expression of miR-148a after knockout、overexpression and knockdown.** (**A)** Genotyping of *miR-148a* WT and *miR-148a* KO mice. **(B)** The expression of miR-148a in BMMs derived from *miR-148a* KO and WT mice. (**C**) Representative images of WT and miR-148a KO female mice at 12 weeks old. (**D-E**) Relative mRNA and protein expression of NRP1 in BMMs which was transfected with NRP1 overexpression lentiviruses. **(F-G)** The silencing efficiency of siRNA in BMMs transfected with siNRP1. (*p < 0.05; **p < 0.01; ***p < 0.001; All data are presented as Mean ± SD).

**Supplementary Fig S2: miR-148a KO attenuates RANKL-induced activation of NF-κB signaling.** **(A)** BMMs were starved for 2 h before being stimulated by RANKL. Then, RANKL (50 ng/mL) was added at the indicated time points (0, 10, 20, 30, and 60 min). Total cellular proteins were collected and detected by Western Blot. **(B)** Quantitative analysis of band intensity of p-P65, IκBα relative to P65, GAPDH; n=3. **(C)** Fluorescence intensity of NFATc1 when NRP1 was knocked down, as determined by IF, Scale bar, 100μm. (*p < 0.05; **p < 0.01; ***p < 0.001, ns= no significance; All data are presented as Mean ± SD).

**Supplementary Fig S3: miR-148a overexpression/inhibition does not affect osteoblastogenesis.** (**A-B)** The expression of miR-148a in BMSCs after transfected with miR-148a agomir/antagomir for 7 or 21 days, n=3 **(C)** Representative images of ALP staining and Alizarin red staining on day 7 and day 21 within BMSCs transfected with miR-148a agomir/antagomir or the corresponding controls. **(D)** Quantitative analysis of ALP-positive cells area and OD values obtained for mineralized matrix solutions in (c). **(E)** Mouse BMSCs were transfected with miR-148a NC/agomir、miR-148a NC/antagomir, and cultured in osteogenic medium for 7 days; RT-qPCR and quantitative analysis were performed to evaluated the mRNA expression of osteoblast-related genes (*Runx2, Alp, Ocn* and *Opn*), n=3. (*p < 0.05; **p < 0.01; ***p < 0.001, ns= no significance; All data are presented as Mean ± SD).

**Supplementary Fig S4: Deletion of miR-148a does not affect osteoblast differentiation and mineralization function *in vitro*. (A)** Mouse BMSCs of miR-148a KO and age-matched WT littermates were cultured with or without osteogenic medium for 7 days. Then the ALP staining was performed; Scale bar, 500μm. **(B)** Quantitative analysis of ALP-positive cells area in (b), n=3. **(C)** Mouse BMSCs of miR-148a KO and age-matched WT littermates were cultured with or without osteogenic medium for 21 days. Then the Alizarin red staining was performed; Scale bar, 500μm. **(D)** Quantitative analysis of the OD values obtained for mineralized matrix solutions in (d), n = 3. **(E)** Real-time PCR analysis of the mRNA levels of *Alp*、*Runx2*、*Opn* and *Ocn* (on day 7) within BMSCs from WT and *miR-148a* KO mice in the osteogenic medium, n=3. (All data are presented as mean ± SD; ns, no significance, compared to the corresponding control group).

**Supplementary Fig S5:** **miR-148a supplementation reverses the bone protection phenotype of miR-148a KO mice. (A)** miR-148a expression level in calvarial tissue of C57BL/6 mice after sham surgery、Ti-particle implantation and miR-148a antagomiR treatment, as detected by qRT-PCR, n=3. **(B)** miR-148a expression level in calvarial tissue of WT and miR-148a KO mice after Ti-particle implantation and miR-148a agomiR treatment, as detected by qRT-PCR, n=3. **(C)** Uterus weight from different groups, n=5. **(D)** miR-148a expression level in tibiae tissue of WT and miR-148a KO mice after OVX surgery and miR-148a agomiR treatment, as detected by qRT-PCR, n=3. **(E)** Representative images of H&E staining from four groups: (WT and KO mice suffered from OVX and supplemented with agomiR-148a and agomiR-NC, respectively). (*p < 0.05; **p < 0.01; ***p < 0.001, ns= no significance; All data are presented as Mean ± SD).

**Supplementary Fig S6:** **GFP and AAV-shNRP1 expression *in vivo*. (A)** Uterus weight from different groups, n=5. **(B)** Fluorescence microscopy was used to monitor GFP expression in frozen sections of mouse tibiae at 6 weeks postsurgery; Scale bar, 500μm. **(C)** Live animal fluorescence imaging was used to monitor GFP expression at 6 weeks postsurgery. **(D)** Representative tibiae images of H&E staining from three groups (WT and KO mice suffered from OVX and injected with PBS, AAV-shNC and AAV-shNRP1, respectively).

**Supplementary Fig S1**

**
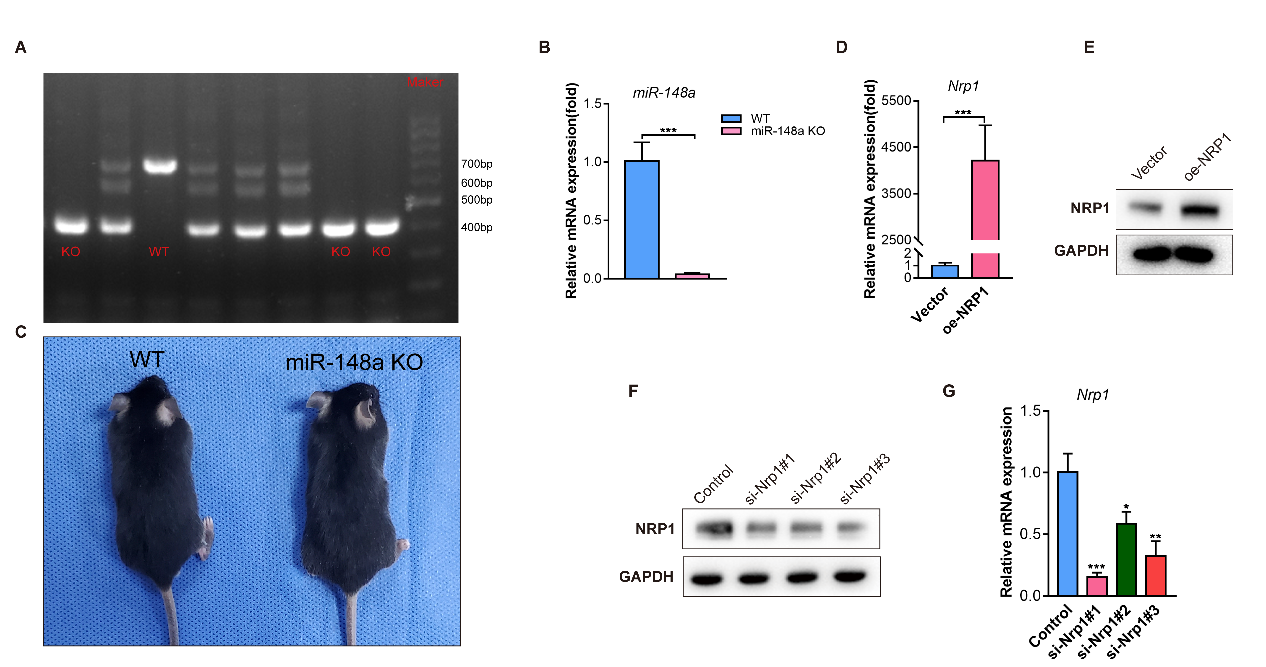
**

**Supplementary Fig S2:**

**
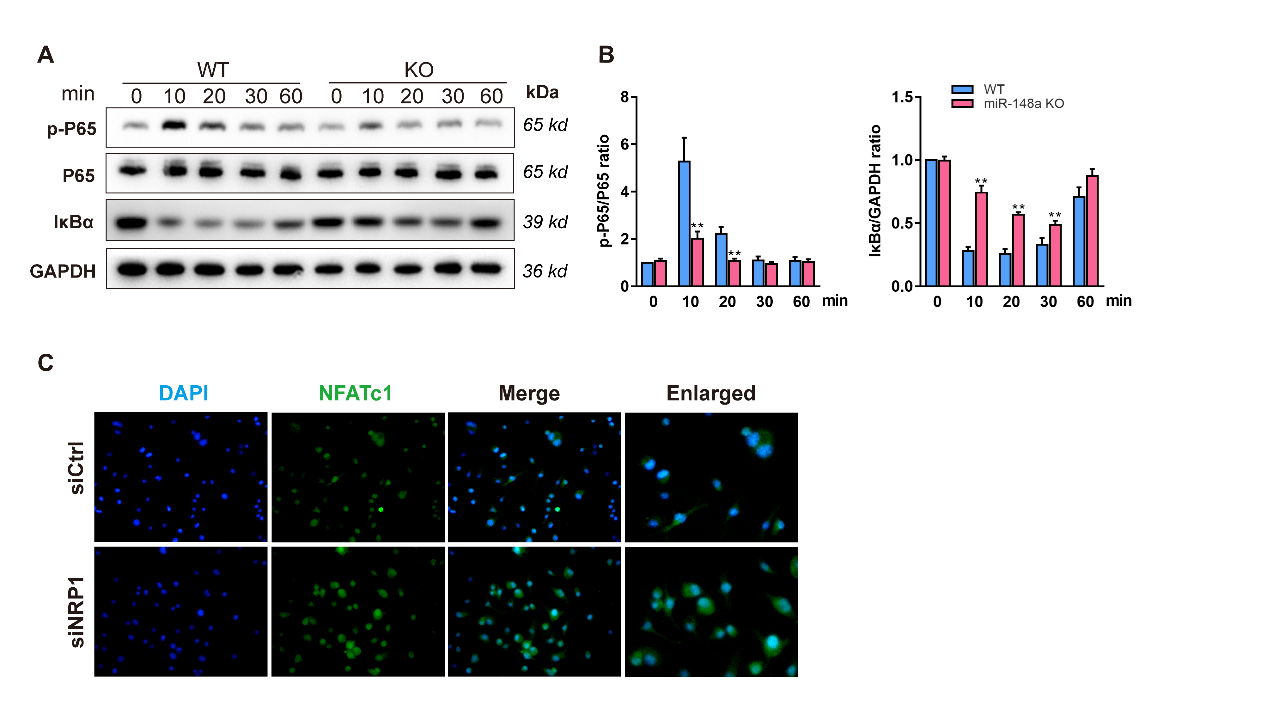
**

**Supplementary Fig S3:**

**
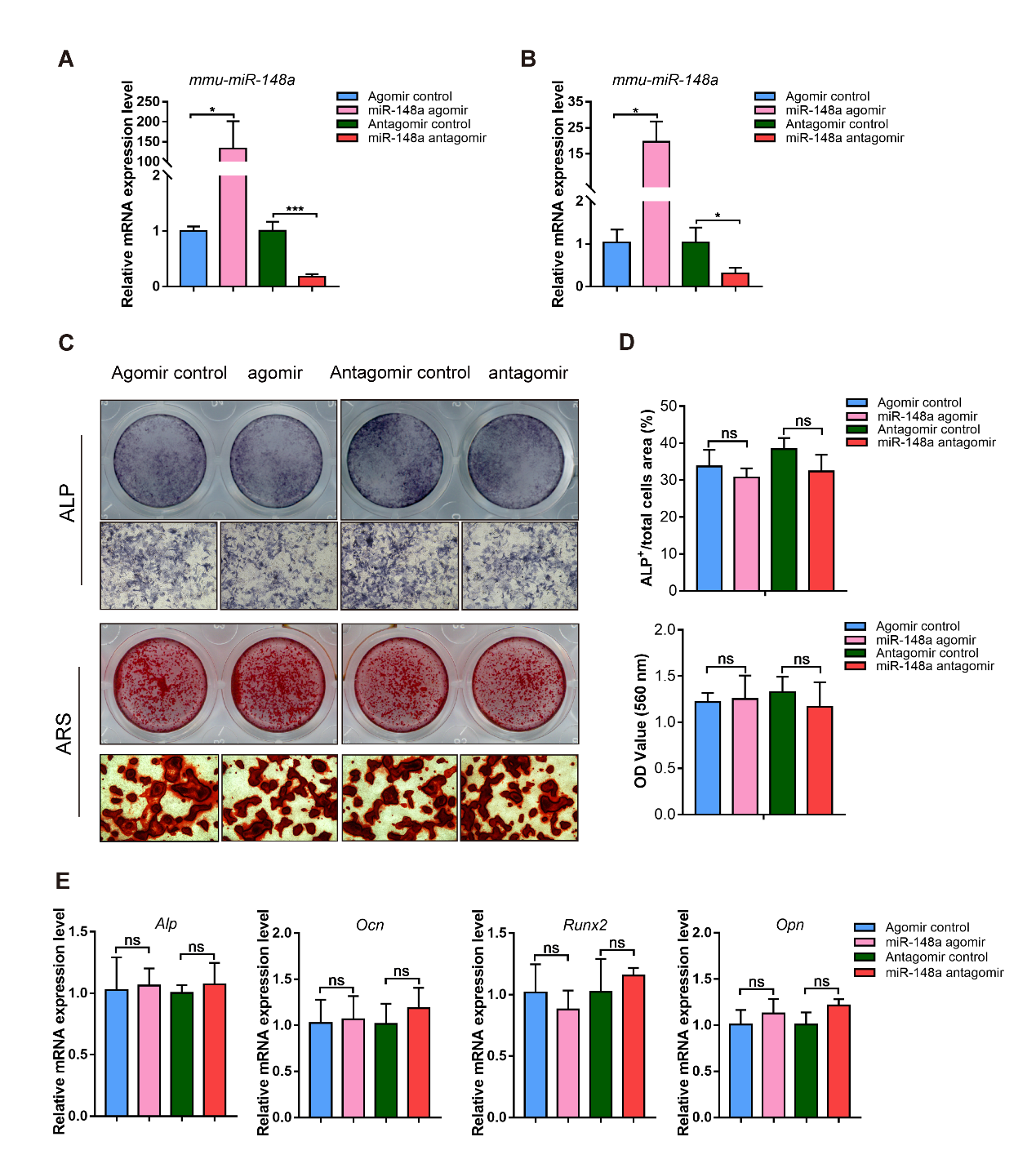
**

**Supplementary Fig S4:**

**
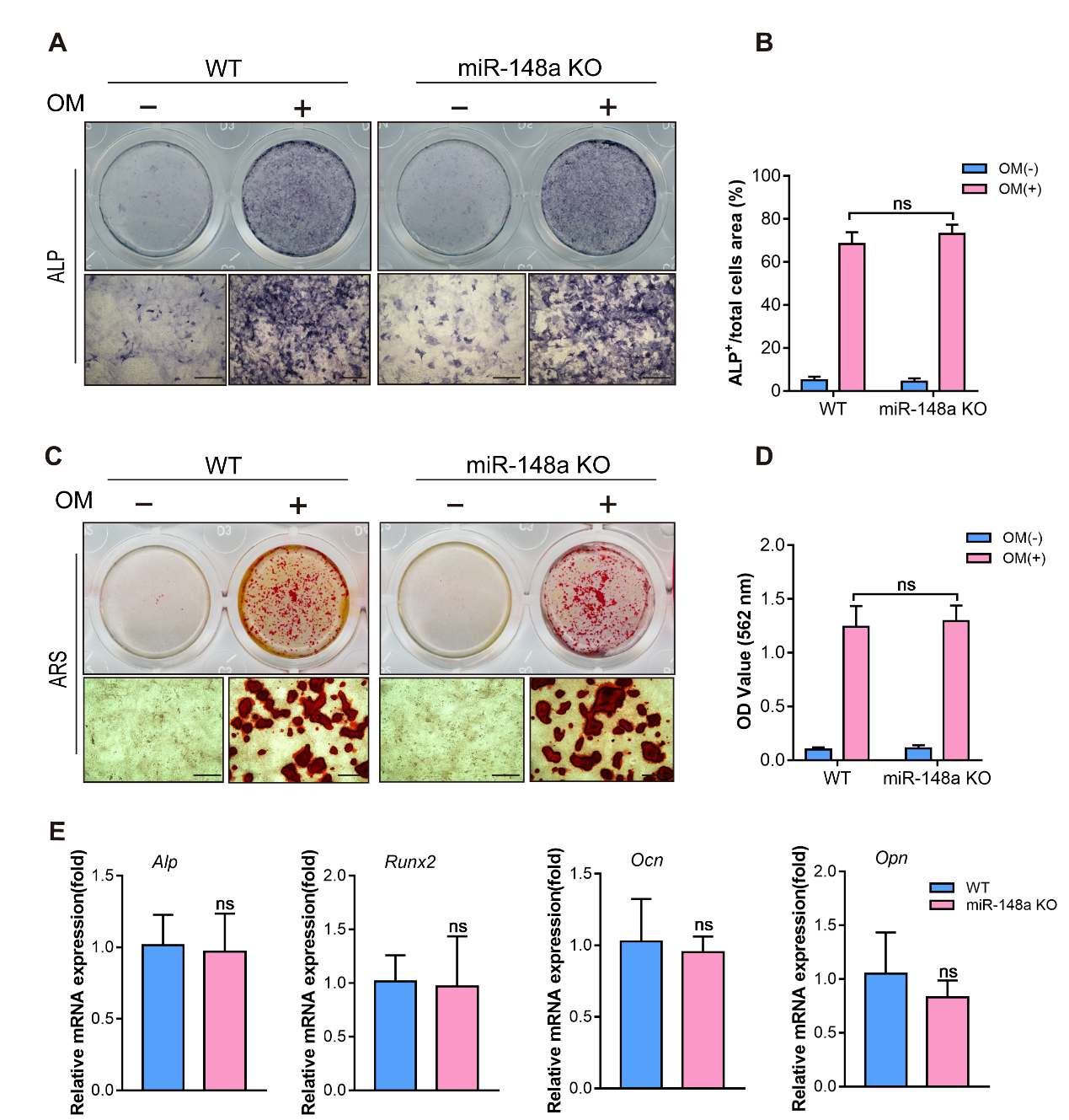
**

**Supplementary Fig S5:**


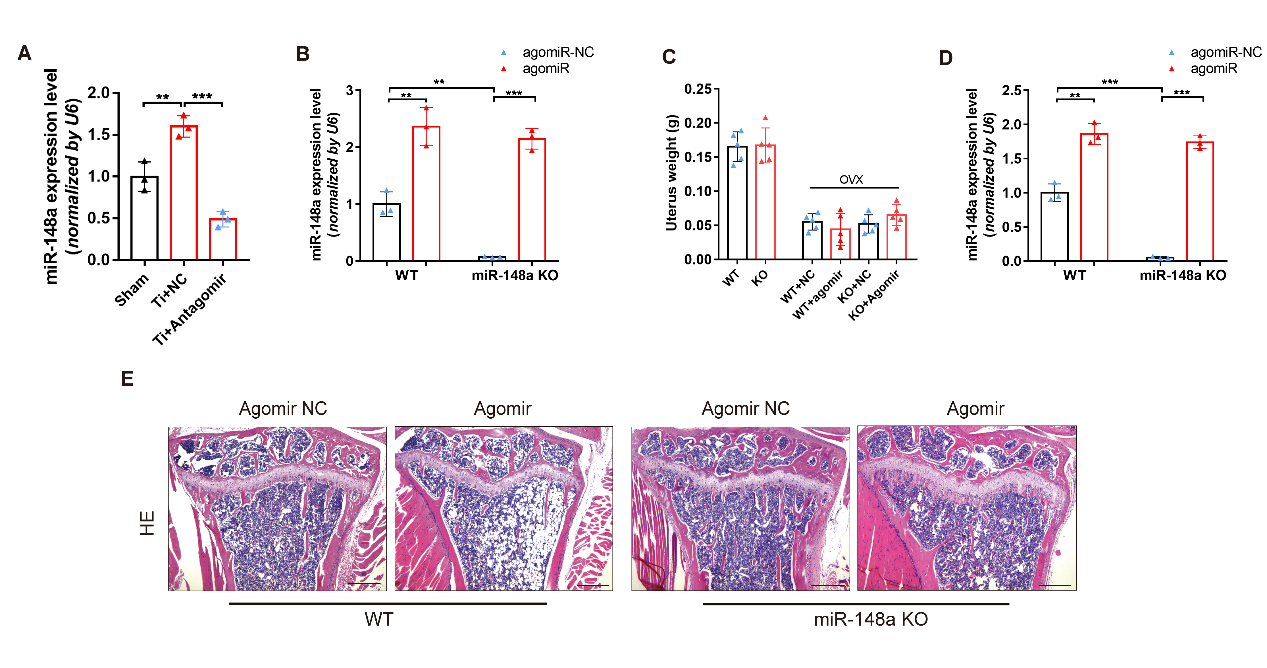


**Supplementary Fig S6:**


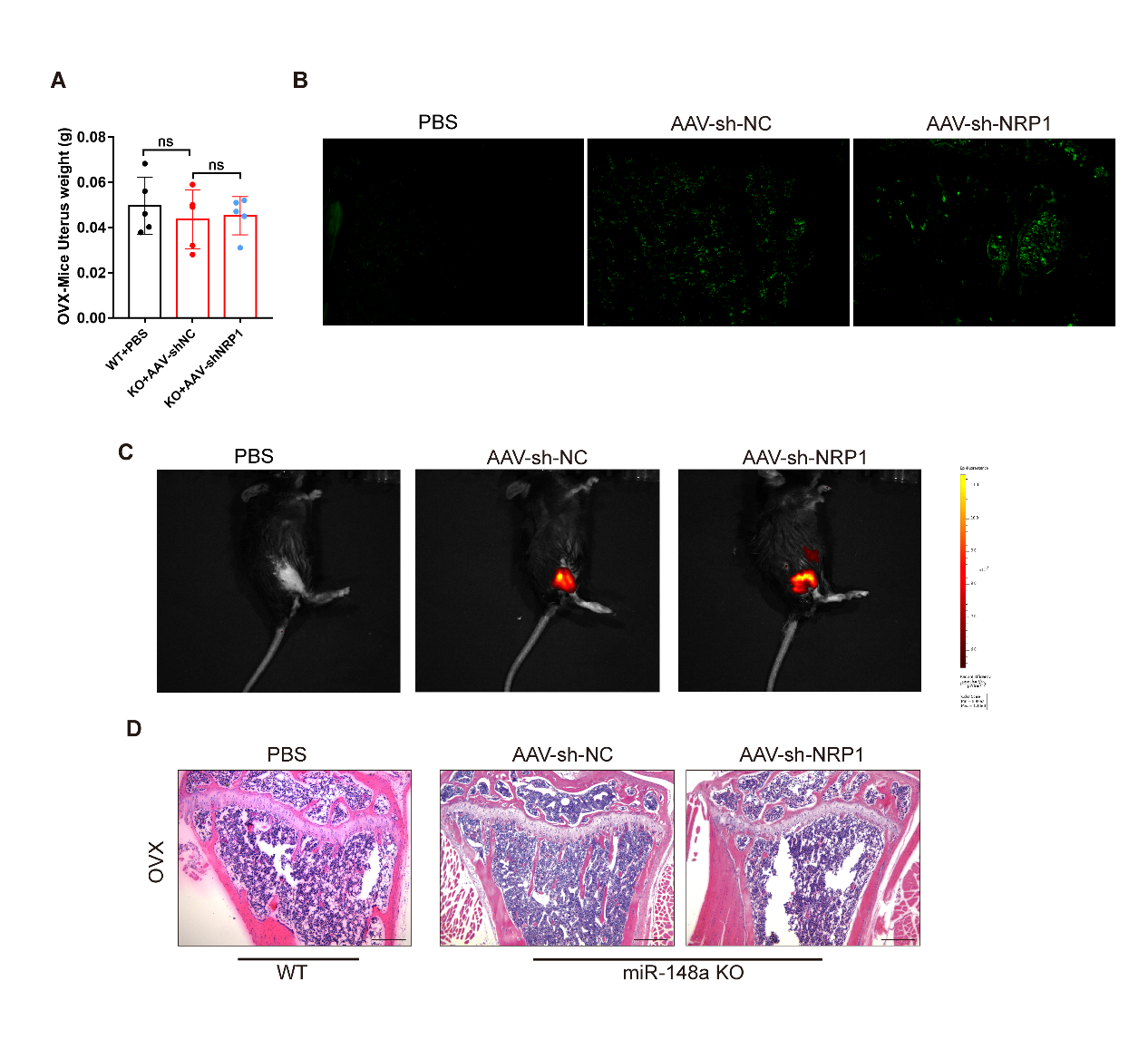

Supplement: Supplementary file 1 — Supplementary Figures [file 41420_2022_1261_MOESM1_ESM.docx]
