# Supplementary material for: MiR-148a deletion protects from bone loss in physiological and estrogen-deficient mice by targeting NRP1
Source: Cell Death Discov. 2022 Nov 29;8:470. doi: 10.1038/s41420-022-01261-5 (PMC9708754; doi:10.1038/s41420-022-01261-5)
Supplement: Supplementary file 2 — Supplementary Tables [file 41420_2022_1261_MOESM2_ESM.docx]

**Supplementary Materials**

**Supplementary Table S1: Primer Sequences**

| **Gene (mouse)** | **Forward primer sequence (5’-3’)** | **Reverse primer sequence(5’-3’)** |
| --- | --- | --- |
| *Nfatc-1* | GGAGCGGAGAAACTTTGCG | GTGACACTAGGGGACACATAACT |
| *c-Fos* | GTTCGTGAAACACACCAGGC | GGCCTTGACTCACATGCTCT |
| *Ctsk* | GAAGAAGACTCACCAGAAGCAG | TCCAGGTTATGGGCAGAGATT |
| *Atp6v0d2* | GTGAGACCTTGGAAGACCTGAA | GAGAAATGTGCTCAGGGGCT |
| *Dc-stamp* | GGGGACTTATGTGTTTCCACG | ACAAAGCAACAGACTCCCAAAT |
| *Trap* | CCATTGTTAGCCACATACGG | CACTCAGCACATAGCCCACA |
| *Gapdh* | AGGTCGGTGTGAACGGATTTG | GGGGTCGTTGATGGCAACA |
| *β-actin* | ACAGCAGTTGGTTGGAGCAA | ACGCGACCATCCTCCTCTTA |
| *Nrp1* | CAGGGTTTTCCATCCGCTATG | ACTCCAGTAGGTGCTGTATAGTT |
| *Opn* | TGGAGAGGTAGAAAAGGCACA | CAAACACACTCTTGGCACCAC |
| *Ocn* | CCCTGAGTCTGACAAAGCCT | GCGGTCTTCAAGCCATACTG |
| *Alp* | TTCATAAGCAGGCGGGGGA | GGTGTACCCTGAGATTCGTCC |
| *Runx2* | GCGCATTCCTCATCCCAGTA | TGGAGTGGATGGATGGGGAT |
| *Lbr* | ATGCCAAGTAGGAAGTTTGTTGA | GATTTGTTGTCGTGGCTCAGA |
| *Arl6ip1* | GTGTTCGCTCGTTGATAACCG | CCCATCGAAGGACTTTGTCAG |
| *Cdk19* | GGCACAGGAATATCTATGTCGG | GCAATGCGATCACATTAGGGT |
| *Naa15* | CATGGAGAAACCCTGGCTATG | TGCCAACACACATGACTCTTT |
| *Cdk5r1* | CTGTCCCTATCCCCCAGCTAT | GGCAGCACCGAGATGATGG |
| *miR-148a Stem-loop primer* | GTCGTATCCAGTGCAGGGTCCGAGGTATTCGCACTGGATACGACACAAAG | |
| *miR-148a* | GCGCGTCAGTGCACTACAGAA | AGTGCAGGGTCCGAGGTATT |
| *U6* | CTCGCTTCGGCAGCACA | AACGCTTCACGAATTTGCGT |

**Supplementary Table S2: Patient information Summary**

**PMOP Patients**

| **Number** | **Sex** | **PMOP** | **BMD (L2)** | **P1NP (ng/ml)** | **β-CTX (pg/ml)** |
| --- | --- | --- | --- | --- | --- |
| P1 | Female | Yes | -2.6 | 69.17 | 363 |
| P2 | Female | Yes | -3.6 | 52.19 | 1008 |
| P3 | Female | Yes | -4.1 | 40.4 | 978 |
| P4 | Female | Yes | -3 | 47.06 | 367 |
| P5 | Female | Yes | -3.2 | 34.61 | 745 |
| P6 | Female | Yes | -2.5 | 44.37 | 288 |
| P7 | Female | Yes | -3.7 | 29.56 | 397 |
| P8 | Female | Yes | -3.2 | 39.75 | 362 |
| P9 | Female | Yes | -4.4 | 37.64 | 502 |
| P10 | Female | Yes | -4.1 | 55.6 | 486 |
| P11 | Female | Yes | -2.9 | 59.17 | 367 |
| P12 | Female | Yes | -2.6 | 25.81 | 455 |
| P13 | Female | Yes | -2.7 | 66.2 | 397 |
| P14 | Female | Yes | -3.8 | 58.03 | 779 |
| P15 | Female | Yes | -2.5 | 14.02 | 177 |
| P16 | Female | Yes | -2.7 | 45.81 | 595 |
| P17 | Female | Yes | -3.2 | 81.27 | 562 |
| P18 | Female | Yes | -4.8 | 40.7 | 812 |
| P19 | Female | Yes | -2.7 | 71.04 | 310 |
| P20 | Female | Yes | -2.6 | 44.32 | 819 |
