# Supplementary material for: MiR-148a deletion protects from bone loss in physiological and estrogen-deficient mice by targeting NRP1
Source: Cell Death Discov. 2022 Nov 29;8:470. doi: 10.1038/s41420-022-01261-5 (PMC9708754; doi:10.1038/s41420-022-01261-5)

Figure 2J

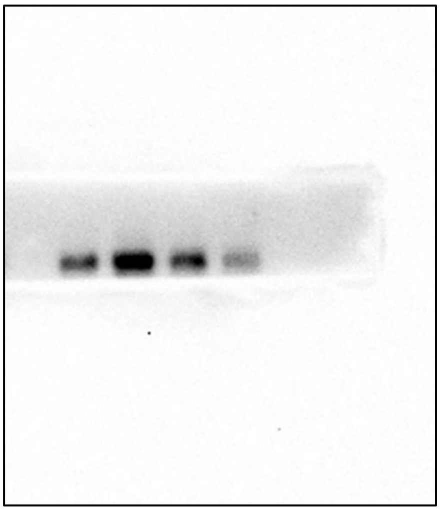

cFos

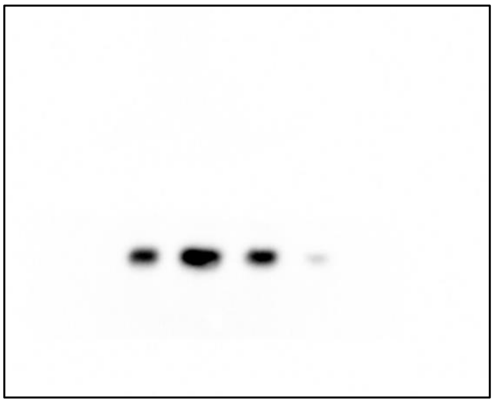

CTSK

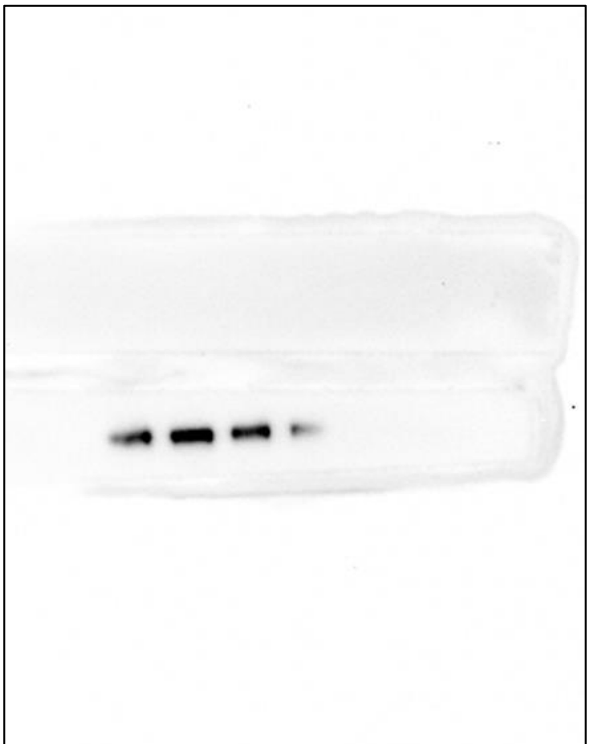

NFATc1

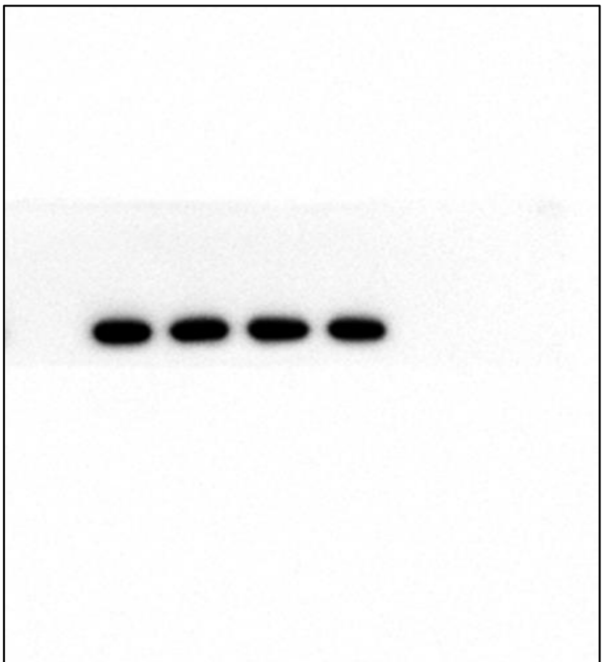

GAPDH

Figure 3H

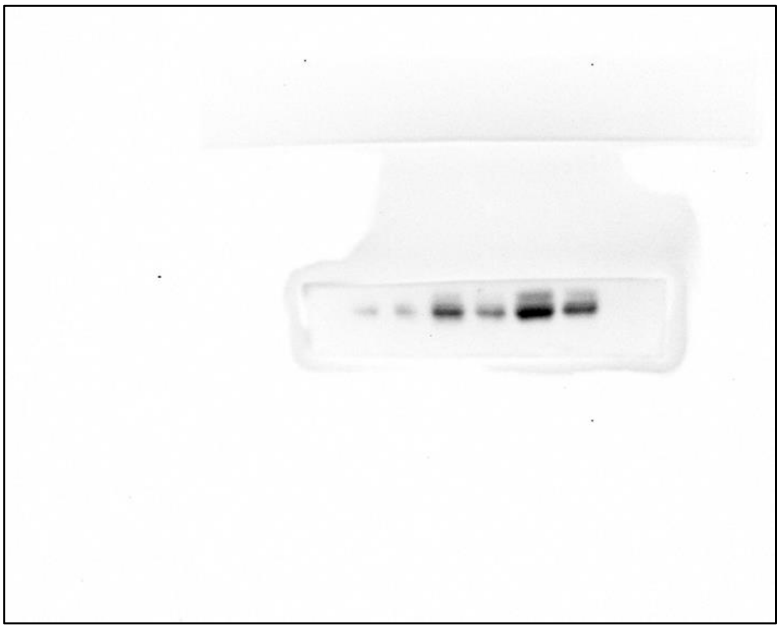

cFos

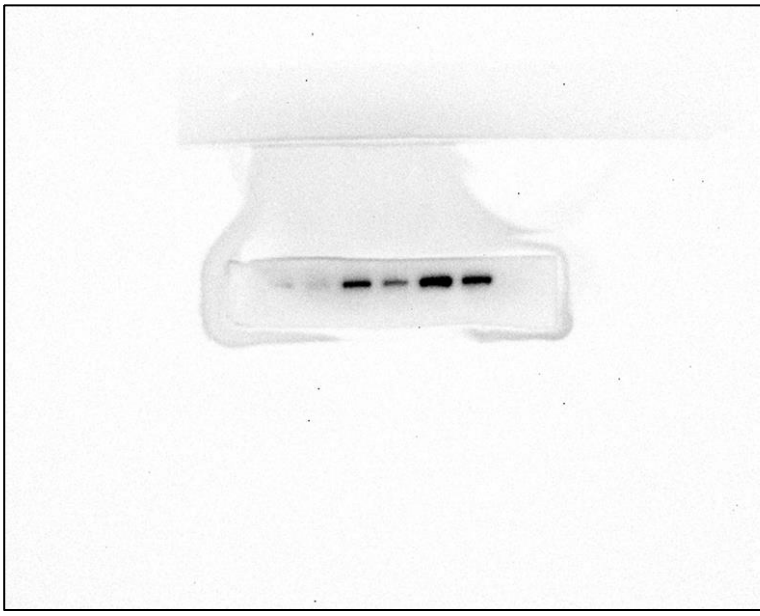

NFATc1

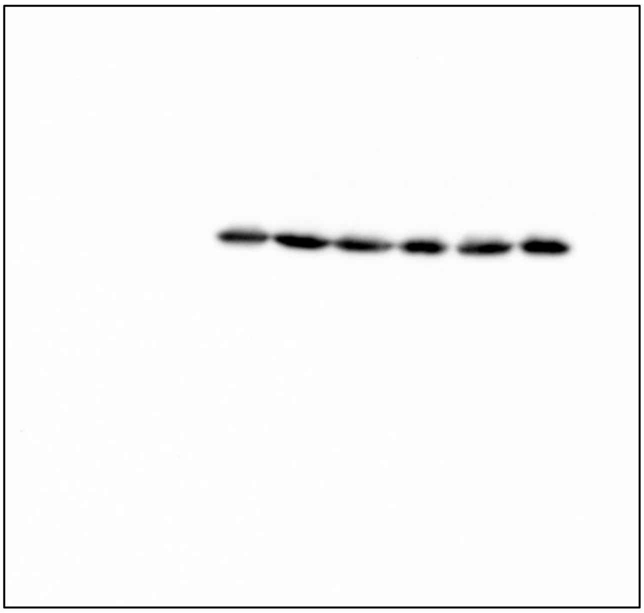

GAPDH

Figure 4G

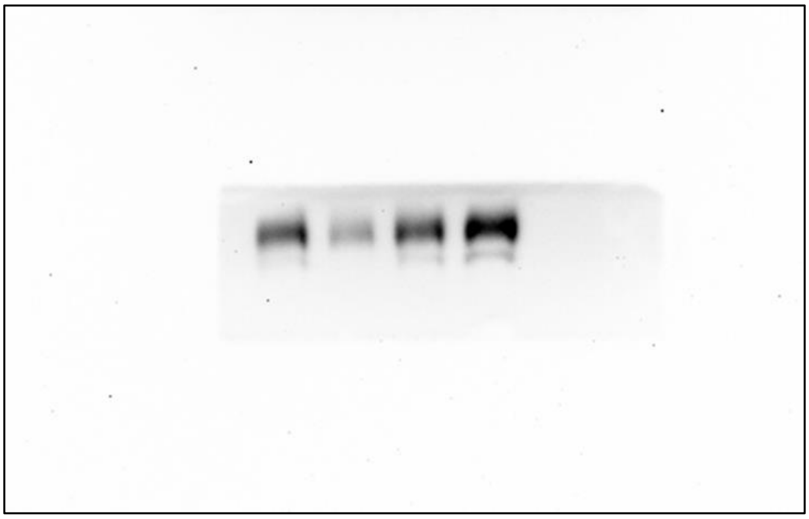

NRP1

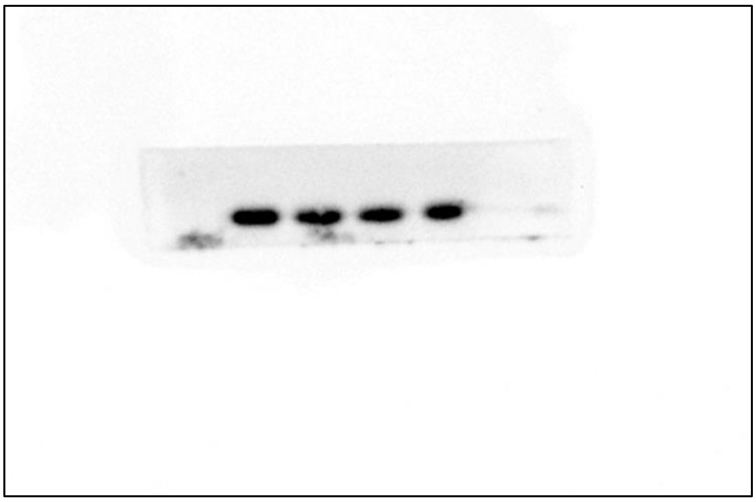

GAPDH

Figure 4H

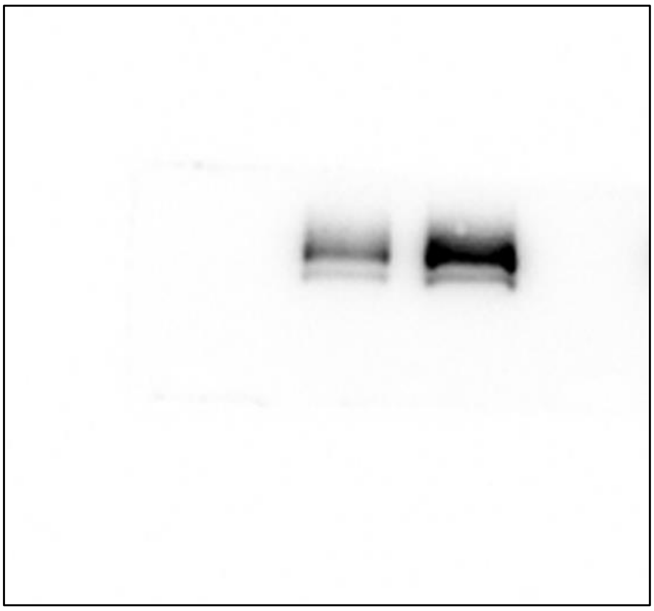

NRP1

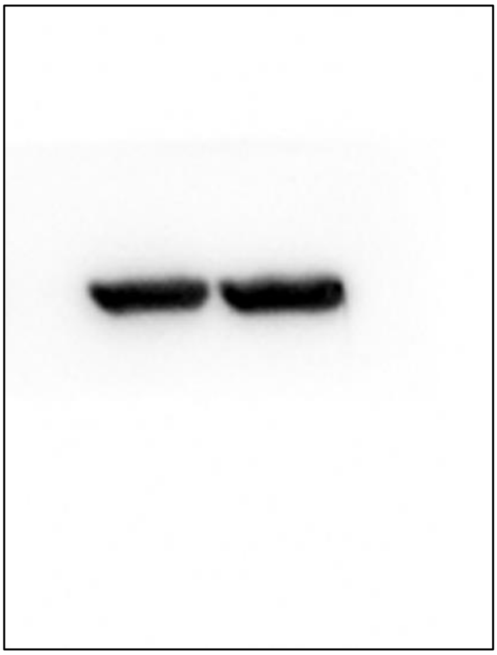

β-actin

Figure 5A

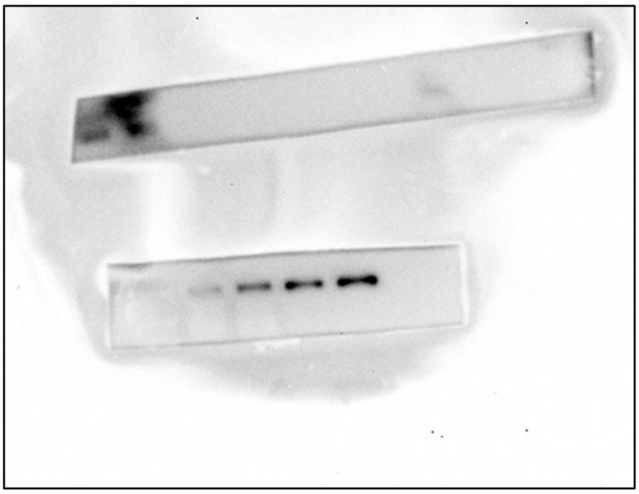

NFATc1

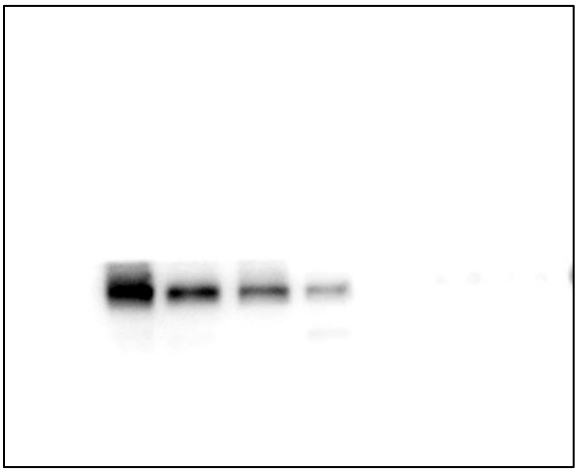

NRP1

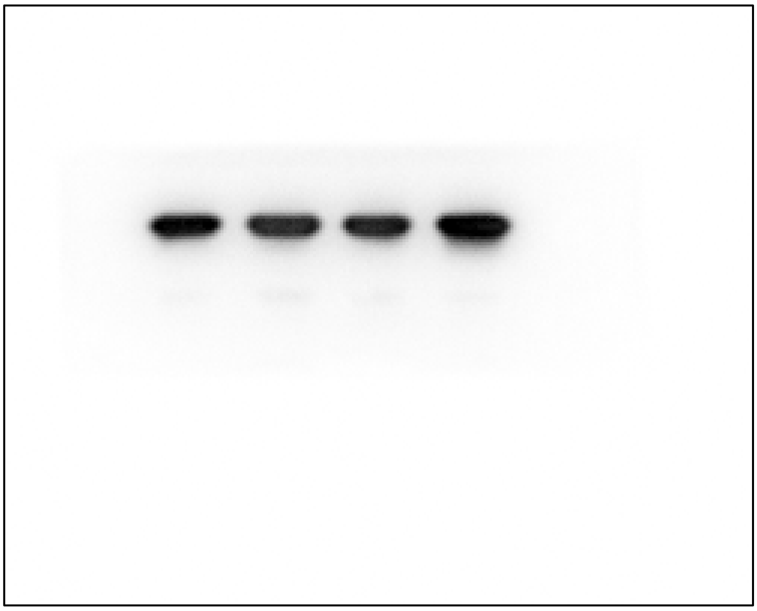

GAPDH

Figure 5E

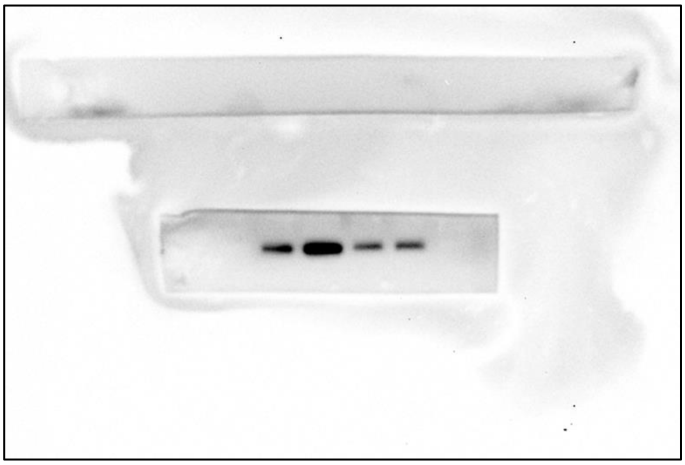

NFATc1

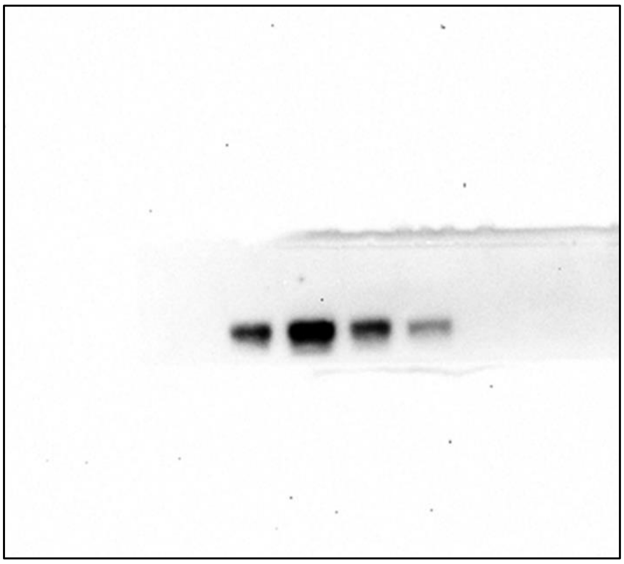

cFos

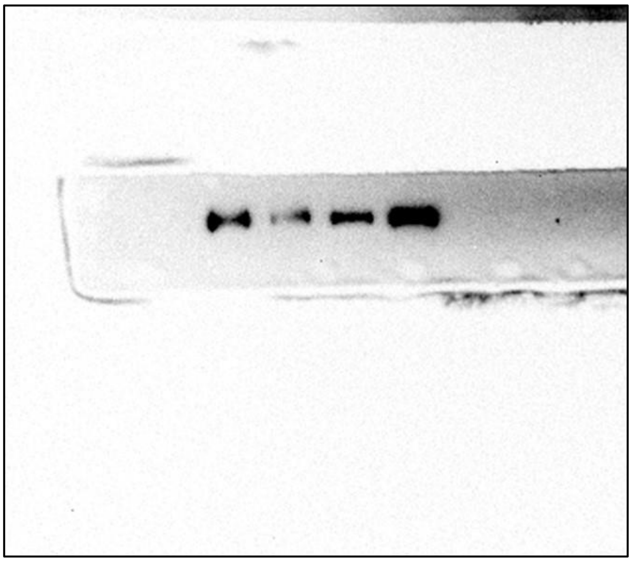

NRP1

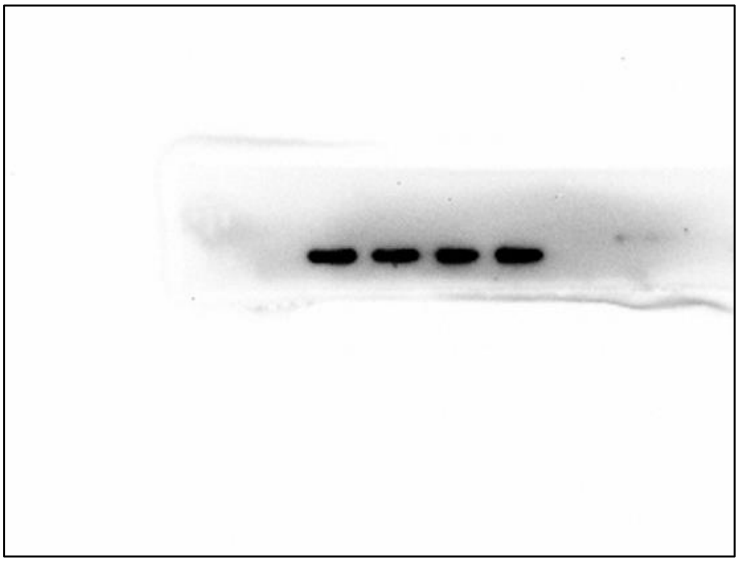

GAPDH

Figure 5K

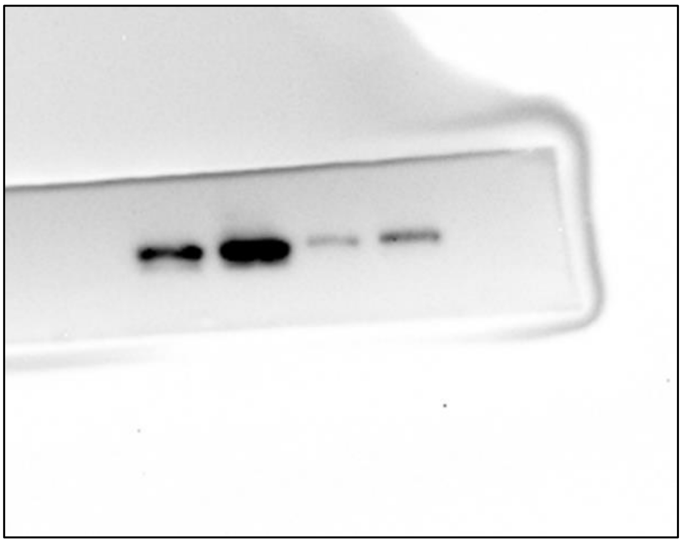

NRP1

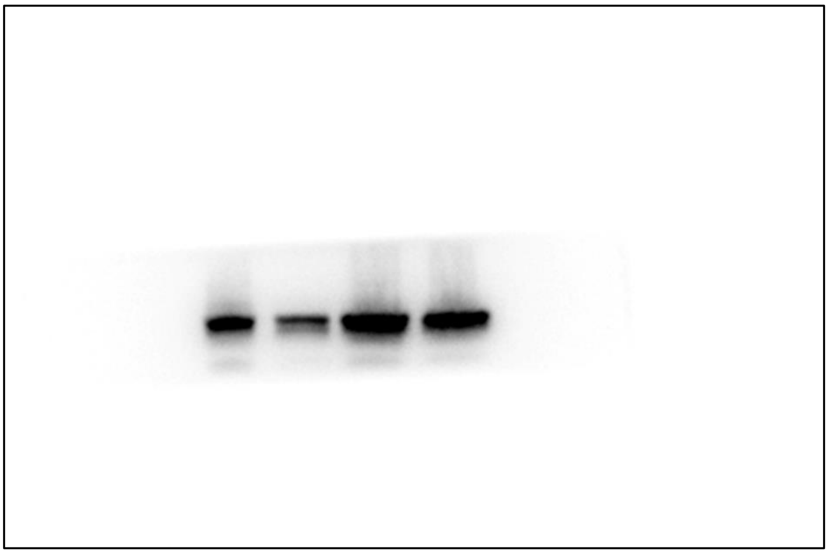

NFATc1

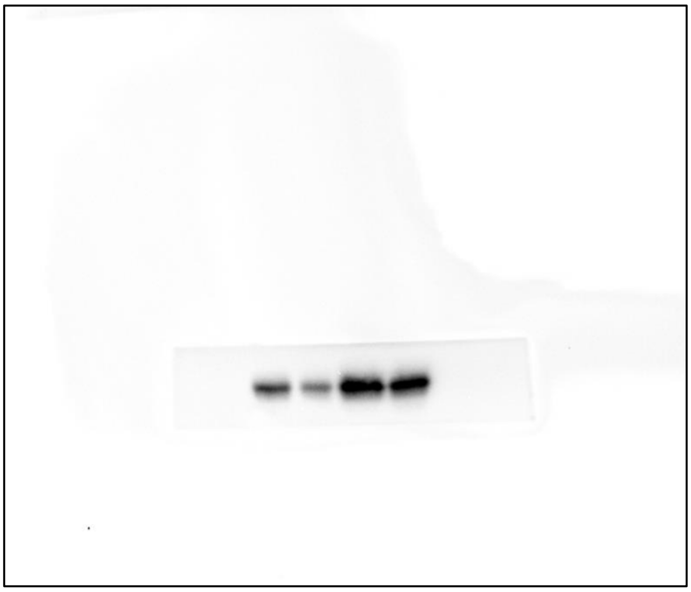

cFos

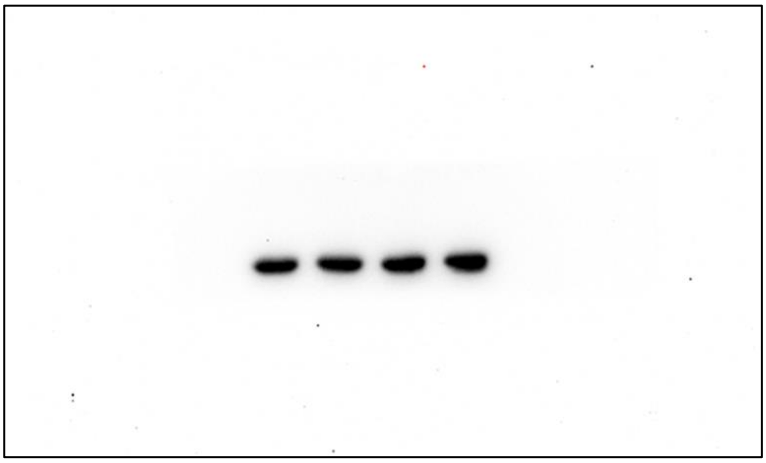

GAPDH

Supplementary S1E

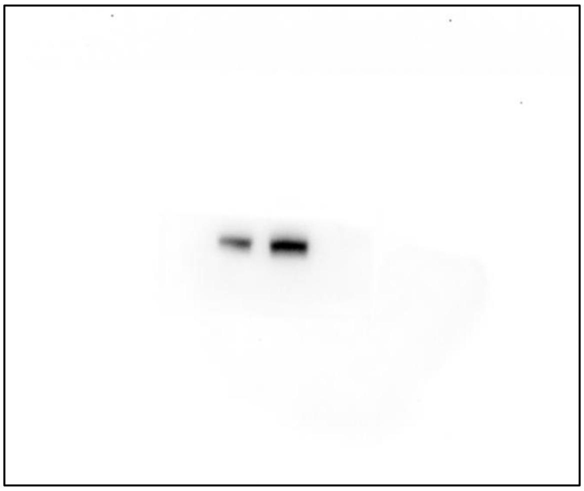

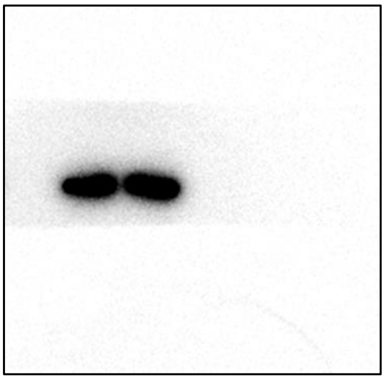

NRP1

GAPDH

Supplementary S1F

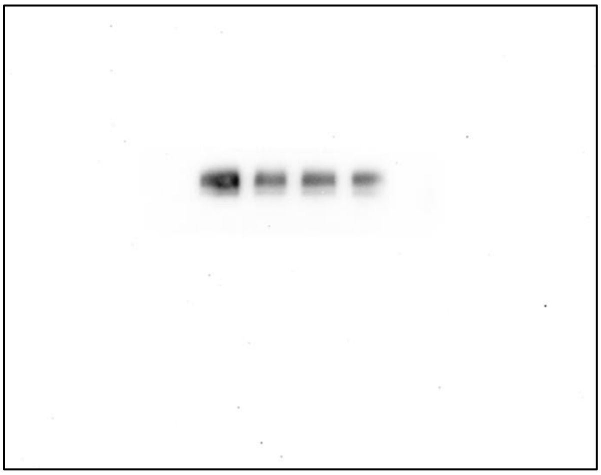

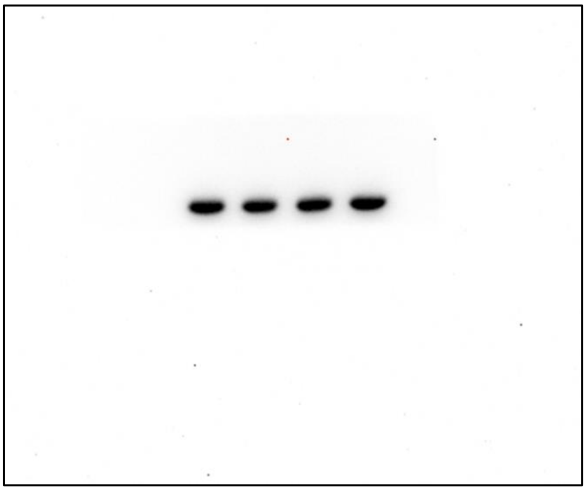

SiNRP1

GAPDH

Supplementary S2A

p-P65

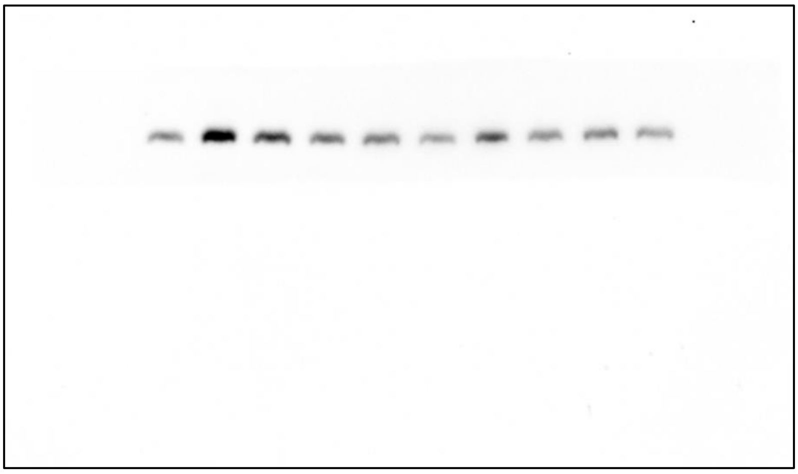

P65

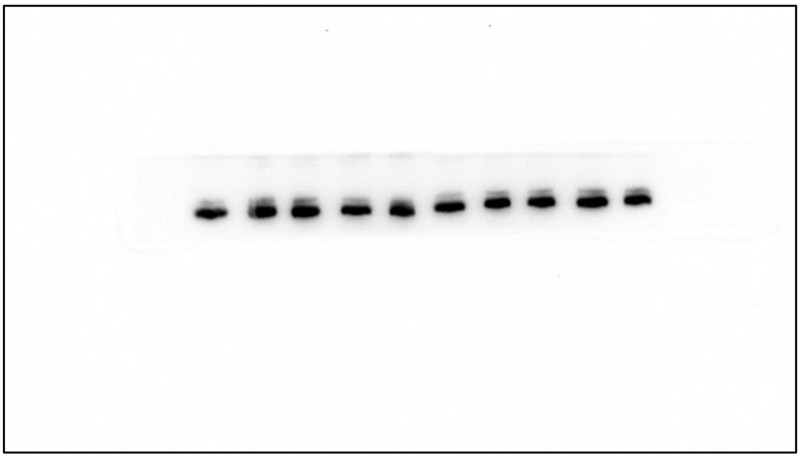

IKBα

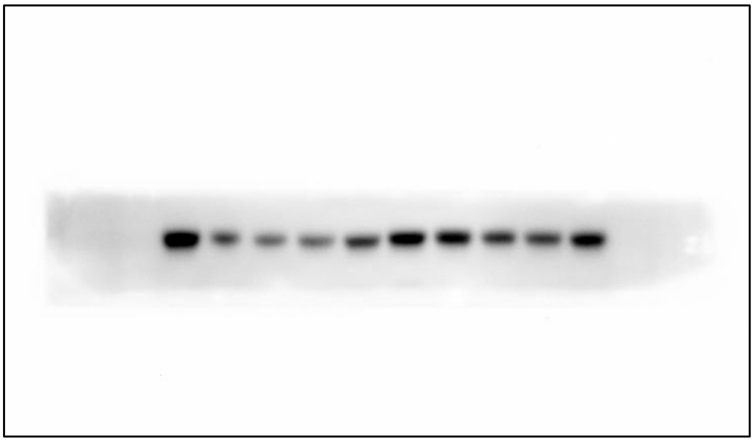

GAPDH

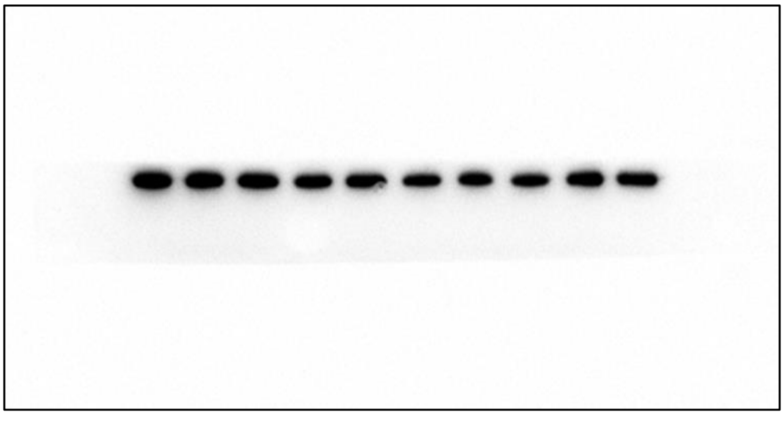

Supplement: Supplementary file 3 — Original data files [file 41420_2022_1261_MOESM3_ESM.pdf]
